# Uncommon Terpenoids from *Salvia* Species: Chemistry, Biosynthesis and Biological Activities

**DOI:** 10.3390/molecules27031128

**Published:** 2022-02-08

**Authors:** Salar Hafez Ghoran, Fatemeh Taktaz, Ali Akbar Mozafari, Murat Tunçtürk, Nazim Sekeroglu, Anake Kijjoa

**Affiliations:** 1 Phytochemistry Research Center, Shahid Beheshti University of Medical Sciences, Tehran, Iran; 2 Medicinal Plant Breeding & Development Research Institute, University of Kurdistan, Sanandaj 66177-15175, Iran; a.mozafari@uok.ac.ir; 3 Department of Biology, Faculty of Sciences, University of Hakim Sabzevari, Sabzevar 96179-76487, Iran; f.taktaz@gmail.com; 4 Field Crop Department, Agricultural Faculty, Van Yuzuncu Yil University, Van 65090, Turkey; murattuncturk@yyu.edu.tr; 5 Phytotherapy, Medicinal and Aromatic Plants Application & Research Center and Biology Department, Faculty of Arts and Science, Gaziantep University, Gaziantep 27310, Turkey; nsekeroglu@gmail.com; 6 ICBAS—Instituto de Ciências Biomédicas Abel Salazar and CIIMAR, Universidade do Porto, Rua de Jorge Viterbo Ferreira, 228, 4050-313 Porto, Portugal

**Keywords:** *Salvia* L., Lamiaceae, sesterterpenoids, dammarane-type triterpenoids, uncommon triterpenoids, cytotoxicity, antiparasitic activity

## Abstract

The search for new bioactive compounds from plant sources has been and continues to be one of the most important fields of research in drug discovery. However, Natural Products research has continuously evolved, and more and more has gained a multidisciplinary character. Despite new developments of methodologies and concepts, one intriguing aspect still persists, i.e., different species belonging to the same genus can produce different secondary metabolites, whereas taxonomically different genera can produce the same compounds. The genus *Salvia* L. (Family Lamiaceae) comprises myriad distinct medicinal herbs used in traditional medicine worldwide that show different pharmacological activities due to the presence of a variety of interesting specialized metabolites, including mono-, sesqui-, di-, sester-, tri-, tetra-, and higher terpenoids as well as phenylpropanoids, phenolic acid derivatives, lignans, flavonoids, and alkaloids. We herein summarize the research progress on some uncommon terpenoids, isolated from members of the genus *Salvia*, which are well recognized for their potential pharmacological activities. This review also provides a current knowledge on the biosynthesis and occurrence of some interesting phytochemicals from *Salvia* species, *viz.* C_23_-terpenoids, sesterterpenoids (C_25_), dammarane triterpenoids (C_30_), and uncommon triterpenoids (C_20_+C_10_). The study was carried out by searching various scientific databases, including Elsevier, ACS publications, Taylor and Francis, Wiley Online Library, MDPI, Springer, Thieme, and ProQuest. Therefore, 106 uncommon terpenoids were identified and summarized. Some of these compounds possessed a variety of pharmacological properties, such as antibacterial, antiviral, antiparasitic, cytotoxic and tubulin tyrosine ligase inhibitory activities. Due to the lack of pharmacological information for the presented compounds gathered from previous studies, biological investigation of these compounds should be reinvestigated.

## 1. Introduction

The genus *Salvia* L., commonly known as “sage”, is one of the largest genera of the family Lamiaceae/Labiatae (subfamily Nepetoideae, tribe Mentheae) comprising over 1000 species worldwide. However, only approximately 150 of them have been investigated [1]. Sage plants are an herbaceous, aromatic and shrubby perennial and are distributed mainly in the Mediterranean, Middle East, the Pacific islands, tropical Africa, and America [2]. Most significant *Salvia* species are found in Mexico (*ca.* 300, which is the largest number) followed by Turkey (with more than 90 and half of which are indigenous) [3], China (*ca.* 84) [4], Iran (*ca.* 62) [5], and Africa (*ca.* 30) [6]. This genus also is rich in medicinal plants with recognized therapeutic properties for treatment of many ailments [6,7].

The most well-recognized species such as *S. officinalis* (common sage), *S. miltiorrhiza* (red sage; Danshen in Chinese), *S. lavandulifolia* (Spanish sage), *S. sclarea* (clary sage), *S. hispanica* (chia), *S. triloba*, and *S. mirzayanii* (a potent anti-diabetic plant in Iran) have been used extensively as medicinal plants in folk medicine in several countries, and some species are cultivated worldwide for culinary purposes [8,9]. Moreover, some species of *Salvia* also exhibit interesting pharmacological activities such as anti-Alzheimer’s and cognitive-enhancing (*S. miltiorrhiza* [10], *S. officinalis* [11], and *S. lavandulaefolia* [12]); antidepressant (*S. sclarea* [13] and *S. elegans* [14]); cardiovascular (*S. hispanica* L. [15]); anti-hyperglycemic/hyperlipidemic (*S. amarissima* Ortega [16], *S. splendens* (scarlet sage) [17], and *S. hydrangea* [18]); hypotensive (*S. cinnabarina* [19]); anti-inflammatory (*S. circinnata* Cav. [20], *S. rosmarinus* Spenn. [21], *S. plebeian* [22], and *S. verbenaca* L. [23]); antioxidant *(S. dracocephaloides* Boiss. [24], *S. multicaulis* [25], *S. pilifera* [26], *S. elegans*, *S. gerggii*, and *S. officinalis* [27]); cytotoxic/anti-proliferative (*S. fruticosa* Mill [28], *S. macrosiphon* Boiss. [29], *S. tebesana* Bunge [30], and *S. atropatana* Bunge [31]); antiprotozoal (*S. clinopodioides* [32], *S. uliginosa* [33], and *S. austriaca* [34]); antifungal (*S. macrochlamys*, *S. recognita* [35], and *S. desoleana* Atzei & Picci [36]); antiviral (*S. plebeia* R. Br. [37], *S. wiedemannii* Boiss. [38], *S. officinalis* [39], *S. dentata* Aiton, *S. scabra* Thunb. [40]); and antimicrobial (*S. euphratica* Montbret, *S. kornenburgii* Rech. f. [41], and *S. chamaedryoides* [42]).

A great diversity of *Salvia* plants, combined with their phytochemical richness, has attracted attention of researchers to discover new pharmacologically active compounds with novel scaffolds. Most significantly, until now, a small number of uncommon terpenoid metabolites have been reported from *Salvia* species, while mono-, sesqui-, di-(abietanes, clerodanes, labdanes, pimaranes, and *ent*-kauranes), sester-, tri- (oleananes, ursanes, and lupanes) terpenoids, phenylpropanoids, phenolic acids, and flavonoids have been frequently reported [2]. 

*Salvia* species are more likely to provide many lead structures that can be exploited as templates for the construction of a variety of compounds with enhanced pharmacological properties and less toxicity. The following subsections of this review will cover C_23_-terpenoids, sesterterpenoids (C_25_), dammarane-type triterpenoids (C_30_), and uncommon triterpenoids (C_20_+C_10_), discussing their structural features as well as biological/pharmacological activities where available.

According to our literature search, there are no comprehensive reviews on uncommon terpenoids from the genus *Salvia* so far. Hence, we present in this review some complementary aspects and an update of the previous reviews [2,43,44], aiming to provide the latest information on their structural diversity and biological activities. The databases used to search for the keywords and terpenoid compounds were PubMed, Web of Science, Scopus, and Google Scholar.

## 2. C_23_-Terpenoids

**Figure 1:**
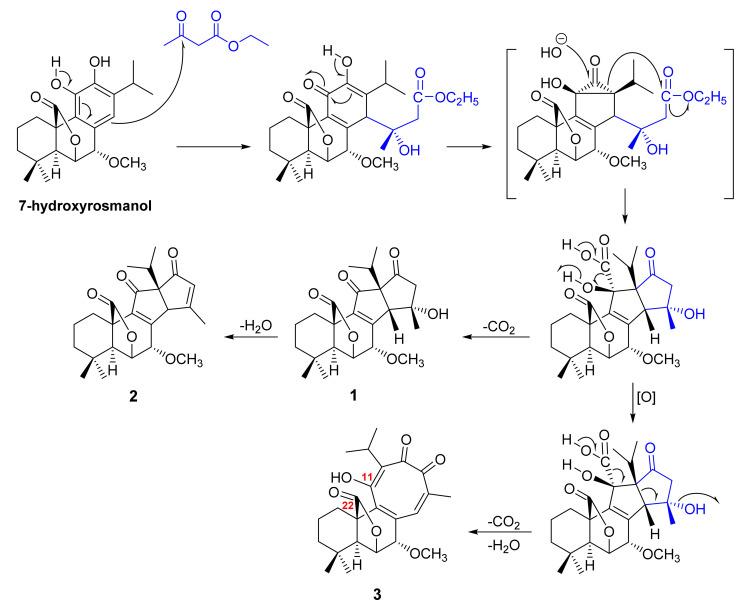
Proposed biosynthetic pathway of **1**–**3**.

Apianane terpenes represent the unprecedented C_23_ basic carbon skeleton with a 6/6/5/5-ring system that originated from tetraprenyl terpenoids, first isolated from the aerial parts of *S. apiana* Jeps (also known as white sage). Phytochemical investigation of *S. apiana* furnished 14-hydroxy-7-methoxy-11,16-diketo-apian-8-en-(22,6)-olide (**1**) and 7-methoxy-11,16-diketo-apian-8,14-dien-(22,6)-olide (**2**) (Figure 1). Luis et al. postulated that the condensation product of 7-methoxyrosmanol with acetoacetyl coenzyme A (CoA) acts as a precursor in the biosynthetic pathway of **1** [45]. Upon reexamination of the same extract, the same research group was able to isolate and identify a new C_23_-hassanane terpenoid that contains a 6/6/8-ring system, 13,14-dioxo-11-hydroxy-7-methoxy-hassane-8,11,15-trien-(22,6)-olide (**3**) (Figure 1). The most interesting ^1^H NMR feature of **3** is the presence of an uncommonly high chemical shift value of a hydroxyl group on C-11 at δ_H_ 17.03. Using this information along with the computational PCMODEL program, it was possible to confirm the presence of a hydrogen bond between the carbonyl group (C-22) and OH-11 [46].

**Figure 2:**
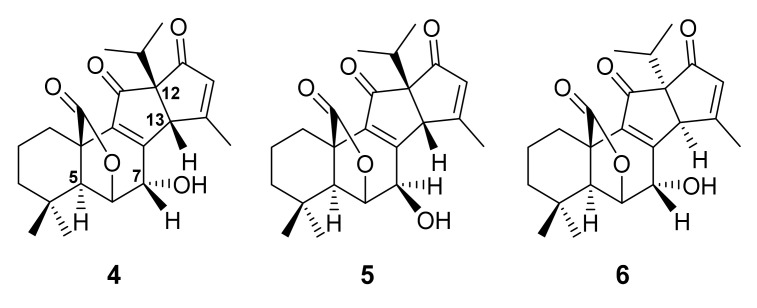
Structures of **4**–**6**.

Repeated chromatographic purification of a dichloromethane (CH_2_Cl_2_) extract of the leaves of *S. officinalis* resulted in the isolation of three apianane terpenes, *viz. rel*-(5*S*,6*S*,7*S*,10*R*,12*S*,13*R*)-7-hydroxyapiana-8,14-diene-11,16-dion-(22,6)-olide (**4**), *rel*-(5*S*,6*S*,7*R*,10*R*,12*S*,13*R*)-7-hydroxyapiana-8,14-diene-11,16-dion-(22,6)-olide (**5**), *rel*-(5*S*,6*S*,7*S*,10*R*,12*R*,13*S*)-7-hydroxyapiana-8,14-diene-11,16-dion-(22,6)-olide (**6**) (Figure 2), together with 15 previously reported compounds. The relative configurations of **4**–**6** were confirmed by a Nuclear Overhauser Effect Spectroscopy (NOESY) from which the presence or absence of correlations from H-5 to H-7, H-7 to H-13, H-13 to 12-isopropyl group was used to determine the α- and/or β-configurations [47].

**Figure 3:**
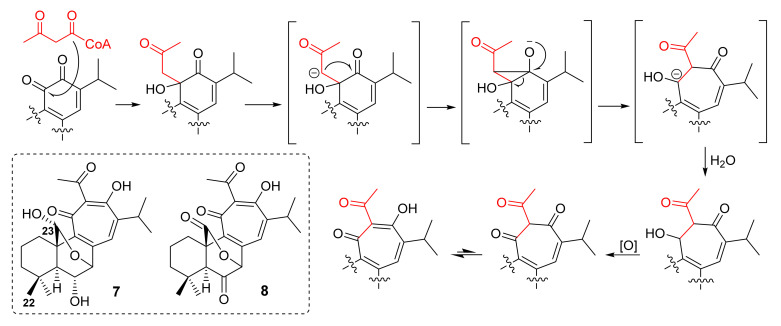
Proposed biosynthetic pathway for **7** and **8**.

Further C_23_-terpenoids with a 6/6/7-ring system, przewalskin A (**7**) and its keto analog (**8**), were isolated from the acetone extract of the aerial parts of *S. przewalskii* Maxim, collected in Shanggelila, Yunnan Province, China. ROESY correlations from Me-22 to H-23 revealed that the hydroxyl group on C-23 in **7** was α-oriented. A putative biosynthetic pathway was also proposed to proceed through a condensation of a normal *o*-quinone diterpene with acetoacetyl-CoA, followed by an intramolecular aldol and oxidation reactions (Figure 3) [48]. Unlike *o*-quinone abietane diterpenes, which exhibited antitumor activity [49], **7** and **8** showed no cytotoxic activity against HL-60 (promyelocytic leukemia), K562 (human immortalized myelogenous leukemia), OVCA-2780 (ovarian), A549 (lung), and HepG-2 (liver) cancer cell lines [48]. 

## 3. Sesterterpenoids

**Figure 4:**
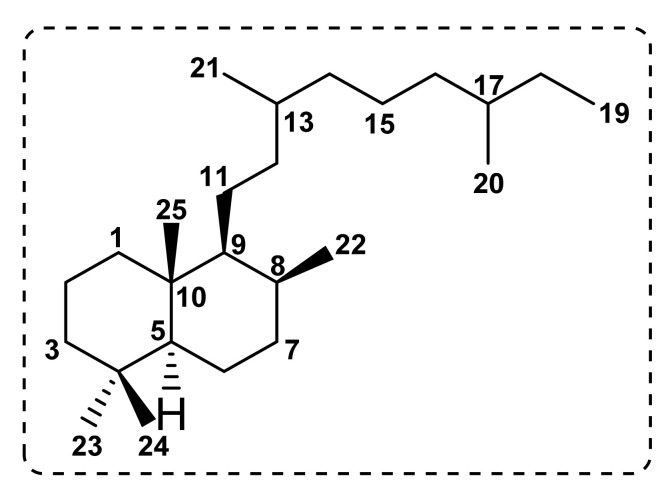
A carbon skeleton of sesterterpenoids from plants of the genus *Salvia*.

Sesterterpenes (C_25_) are a class of terpenoid compounds that have been frequently reported from bacteria, fungi, lichens, insects, marine invertebrates (particularly marine sponges), and some higher plant families such as Asteraceae, Lamiaceae, Lobariaceae, Gentianaceae, and Pteridaceae. Sesterterpenoids are among the most interesting classes of specialized metabolites since they possess relevant biological and pharmacological activities [50]. Sesterterpenoids reported from plants of the genus *Salvia* possess a prenyllabdane skeleton (Figure 4) [51], all containing a γ-lactone ring. Accordingly, these specialized metabolites are divided into three different subgroups.

### 3.1. Bicyclic Sesterterpenoids

**Figure 5:**
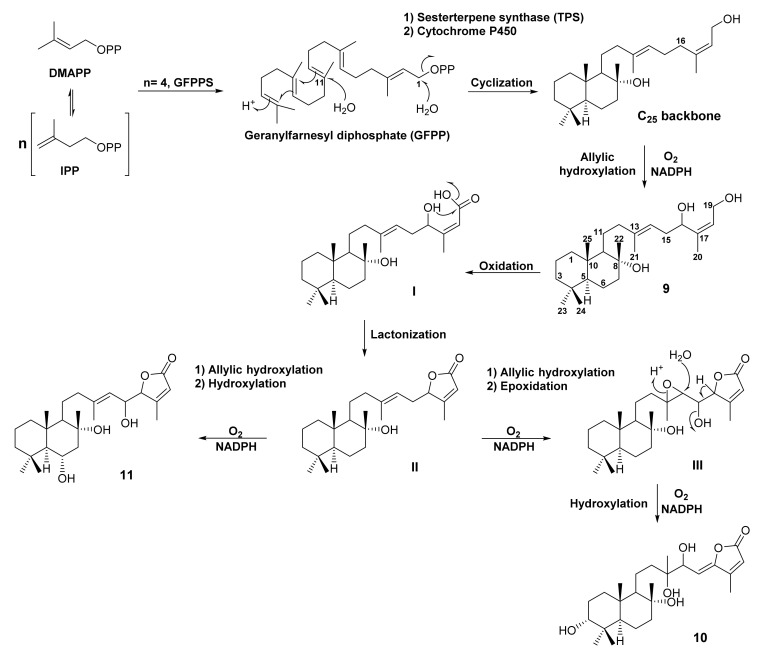
Structures of **9**–**11** and their proposed biosynthetic pathway.

A literature search on *Salvia* plants revealed that only eight species biosynthesize γ-lactone-containing bicyclic sesterterpenes. They are *S. hypoleuca*, *S. syriaca*, *S. sahendica*, *S. mirzayanii*, *S. palaestina*, *S. lachnocalyx*, *S. dominica* and *S. tingitana*, half of which are endemic to Iran. A plausible biosynthetic pathway is shown in Figure 5. As an example of this type of sesterterpenes, we can infer the following specialized metabolites with the increasing oxidation state of C-23 from CH_3_ to CH_2_OH, CHO, COOH, COOMe and the lactone ring between C-6 and C-23.

#### 3.1.1. Bicyclic Sesterterpene Lactones Containing C-23 Methyl Group

Purification of the *n*-hexane-insoluble portion of a CH_2_Cl_2_ extract of fresh aerial parts of *S. tingitana*, obtained in San Remo, Italy, by several chromatographic techniques including column chromatography (CC) of silica gel and Sephadex LH-20, and semi-preparative high performance liquid chromatography (HPLC), resulted in the isolation of (13*E*)-labd-13(14),17(18)-dien-8α,16,19-triol (**9**) (Figure 5). The structure of **9** was elucidated by high-resolution electrospray ionization mass spectrometry (HRESIMS) and 1D and 2D NMR spectral analysis, while its relative configuration was established by NOESY correlations from H-5 to H-9, H_3_-22 to H_3_-24 and H_3_-25 as well as the values of coupling constants between H-14 and H-18 [52]. 

A polyhydroxy sesterterpenoid, 3α,8α,13,14-*erythro*-tetrahydroxy-labd-15,17-dien-16,19-olide (**10**) (Figure 5), was isolated from the acetone extract of the aerial parts of *S. palaestina* Bentham, collected in Jordan. The structure of **10** was established based on extensive analysis of 1D and 2D NMR spectra and HRMS data. The stereochemistry of C-13/C-14 diol was determined as *erythro* by observation of the ^1^H NMR signals of the acetonyl methyl groups of its acetonide derivative, which appeared as two separated three-proton singlets at δ_H_ 1.29 and 1.56 [53]. Another C-23 methyl sesterterpene, 6α,8α,15*S*-trihydroxy-labd-13(14),17-dien-16*S*,19-olide (**11**) (Figure 5), was isolated from the CHCl_3_-MeOH extract of the aerial parts of *S. dominica* L., collected at As-Subayhhi, in Al-Balqa Province, Jordan, by a Sephadex LH-20 column chromatography, solid-phase extraction (SPE) and purified by reversed-phase HPLC. The structure of **11** was established by 1D and 2D NMR spectral analysis and HRMS data [54].

Investigation of the *Arabidopsis* genome revealed that the putative sesterterpene gene clusters consist of geranylfarnesyl diphosphate synthase (*GFPPS*), terpene synthase (*TPS*), and cytochrome *P450s*. Therefore, the functional identification of *GFPPS-sesterTPS-P450* gene clusters of *Arabidopsis* in vitro, and subsequent detection of sesquiterpenes *in planta* were achieved. Furthermore, subcellular localization of identified enzymes involved in sesterterpene biosynthesis suggested that sesterterpenes (GFPPSs and sesterTPSs) are produced from the plastidial 2-C-methyl-D-erythritol 4-phosphate (MEP) pathway. Due to the presence of *GFPPS-TPS-P450* clusters in the *Arabidopsis* genome, the TPSs located in these biosynthetic gene clusters were investigated to verify if they utilize GFPP as a substrate to produce sesterterpene backbones (even though no sesterterpene has been reported previously in *Arabidopsis*). By using different expression systems, 18 Brassicaceae-specific sesterTPSs were characterized, and 20 sesterterpene products were purified and elucidated. Moreover, phylogenetic analysis of plant TPS sequences clearly showed that functional sesterTPSs evolved from the TPS-a subfamily, the members of which always utilize GPP and/or FPP as substrates. However, since plant sesterTPSs have been identified only from Brassicaceae, it is still unclear whether the sesterTPSs from other plant species, such as Lamiaceae, evolved from the TPS-a subfamily or from other TPS subfamilies [50]. 

Based on this finding, **9** is hypothesized to be generated by allylic hydroxylation at C-16 of the side chain of the bicyclic sesterterpene skeleton formed by cyclization of GFPP. Oxidation of the hydroxyl group at C-19 gives the intermediate **I**, which undergoes lactonization between OH-16 and the carboxylic acid at C-19 to give **II**. The intermediate **II** can proceed in two directions. First, hydroxylation at C-6 and C-15 leads to the formation of **11**. On the other hand, hydroxylation at C-15 and epoxidation of the double bond between C-13 and C-14 produces **III**. Hydrolysis of the 13,14-epoxide and dehydration of OH-15 in **III** gives rise to **10** (Figure 5).

#### 3.1.2. Bicyclic Sesterterpene Lactones Containing C-23 Hydroxymethyl Group

**Figure 6:**
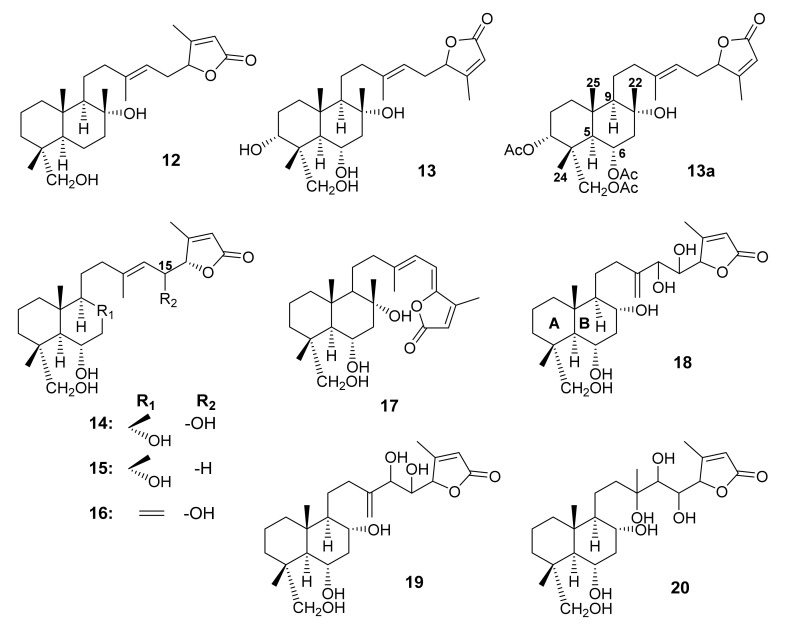
Structures of **12**–**20**.

By using CC of silica gel, followed by Sephadex LH-20 and semi-preparative HPLC, (13*E*)-8α,23-dihydroxy-labd-13(14),17(18)-dien-16,19-olide (**12**) (Figure 6) was isolated from a defatted fraction of CH_2_Cl_2_-soluble extract of *S. tingitana*, collected in San Remo, Italy. The structure of **12** was established by HRESIMS data, and 1D and 2D NMR spectral analysis. The relative configuration of **12**, determined by NOESY correlations, was identical to that of **9** [52].

Salvisyriacolide (**13**) (Figure 6), a polar sesterterpene lactone with four hydroxyl groups, was isolated from the MeOH-soluble fraction of *S. syriaca* L., collected in the North of Taleghan, Iran. The structure of **13** was established by extensive NMR spectral analysis. In order to determine the relative configuration, **13** was acetylated to give its triacetate (**13a**), which exhibited nuclear Overhauser effects (NOE) between H-24 and H-6 (8%), H-25 and H-6 (10%), H-9 and H-5 (8%), H-22 and H-6 (8%), as well as H-22 and H-25 (8%) [55].

Dal Piaz et al., in their screening program for tubulin-tyrosine ligase (TTL) inhibitors, have identified a series of C-23 hydroxymethyl sesterterpenoids from the aerial parts of *S. dominica* L., obtained at As-Subayhhi in Al-Balqa Province, Jordan. By using a Sephadex LH-20 column chromatography, SPE, and reversed-phase HPLC, the CHCl_3_-MeOH fraction furnished the following sesterterpene lactones: 6α,8α,15*S*,23-tetrahydroxy-labd-13(14),17-dien-16*S*,19-olide (**14**), 6α,8α,23-trihydroxy-labd-13(14),17-dien-16*R*,19-olide (**15**), 6α,15*S*,23-trihydroxy-labd-8(22),13(14),17-trien-16*S*,19-olide (**16**), 6α,8α,23-trihydroxy-labd-13(14),15,17-trien-16,19-olide (**17**), 6α,8α,23,14,15-*threo*-pentahydroxy-labd-13(21),17-dien-16,19-olide (**18**), and 6α,8α,23,14,15-*erythro*-pentahydroxy-labd-13(21),17-dien-16,19-olide (**19**) (Figure 6). The structures of **14**–**19** were established by 1D and 2D NMR spectral analysis and ESI-MS. The relative configurations of C-4, C-5, C-6, C-8, C-9, and C-10 in **14** were determined based on coupling constant values for H-5, H-6, and H-9, and their α orientation was established by ROESY correlations from H-9 to H-5, Me-22 to Me-24 and Me-25. Moreover, the absolute configuration of C-15 was determined as *R* by a modified Mosher’s method. The stereochemistry of rings A and B of **18** and **19** were identical to that of **14**. In order to determine the relative configurations of C-14 and C-15 in **18** and **19**, their acetonides were obtained. The ^1^H NMR spectrum showed that both the acetonyl methyl groups in **18** appeared as one singlet at ca. δ_H_ 1.31, while those of **19** appeared as two singlets at δ_H_ 1.30 and 1.56, indicating that the relative configurations of C-14 and C-15 in **18** and **19** are *threo* and *erythro*, respectively [54]. The same authors have further investigated the defatted acetone extract of *S. dominica* using liquid chromatography coupled with mass spectrometry (LC-MS). In addition to 6α,8α,13,23,14,15-*threo*-hexahydroxy-labd-17-en-16,19-olide (**20**) (Figure 6), **14**–**19** were identified in a single step. Compound **20** was identified by MS and MS/MS fragmentations. The main characteristic MS pattern of **20** displayed a sodium adducted ion peak [M+Na]^+^ at *m*/*z* 493, and its collision-induced fragmentation generated fragment ion at *m*/*z* 463 (-30 amu), indicating that CH_2_OH is linked to C-4. Moreover, a product ion at *m*/*z* 249 [C_11_H_14_O_5_+Na]^+^ was observed, suggesting a side chain carrying three hydroxyl groups [56].

#### 3.1.3. Bicyclic Sesterterpene Lactones Containing C-23 Formyl Group

**Figure 7:**
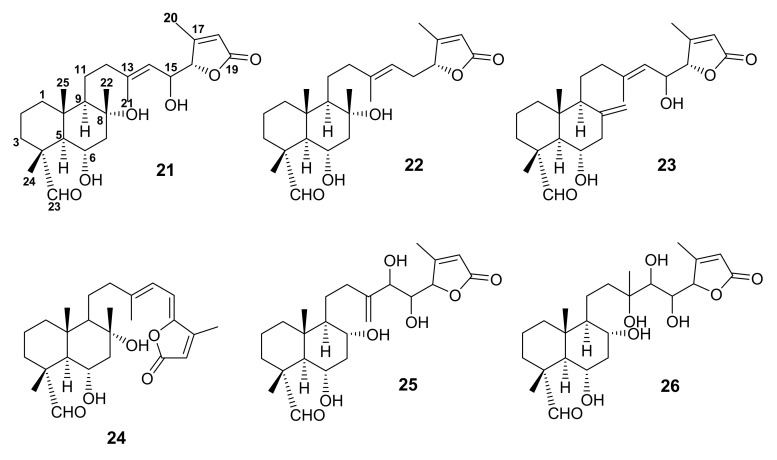
Structures of **21**–**26**.

A series of C-23 formyl bicyclic sesterterpene lactones, including 6α,8α,15(*S*)-trihydroxy-23-oxo-labd-13(14),17-dien-16*S*,19-olide (**21**), 6α,8α-dihydroxy-23-oxo-labd-13(14),17-dien-16*R*,19-olide (**22**), 6α,15(*S*)-dihydroxy-23-oxo-labd-8(22),13(14),17-trien-16*S*,19-olide (**23**), 6α,8α-dihydroxy-23-oxo-labd-13(14),15,17-trien-16,19-olide (**24**), 6α,8α,14,15-*threo*-tetrahydroxy-23-oxo-labd-13(21),17-dien-16,19-olide (**25**), and 6α,8α,13,14,15-*threo*-23-oxo-pentahydroxy-labd-17-en-16,19-olide (**26**) (Figure 7) was reported from the CHCl_3_-MeOH extract of the aerial parts of *S. dominica* L. The structures of **21**–**26** were established by 1D and 2D NMR spectral analysis and ESIMS data. In the case of **21**, 1D-ROESY experiments established the α-orientation of CHO-23, i.e., irradiation of Me-24 at δ_H_ 1.18 increased the intensity of Me-25 and Me-22 signals. Comparison of the NMR spectral data of **24** with those of **14** and **17** revealed that **24** and **17** have the same side chain but differ in the bicyclic moiety, whereas the bicyclic moiety of **14** and **17** are identical. The structure of **26** was proposed based on the presence of [M+Na]^+^ at *m*/*z* 491, indicating its molecular mass of 468 amu, which is 2 amu less than the molecular mass of **20**. Like **20**, its MS/MS spectrum showed signals at *m*/*z* 393 and *m*/*z* 249, indicating that **26** has the same side chain as **20**. Moreover, the signal at *m*/*z* 401 was indicative of the presence of five eliminable hydroxyl groups. On the other hand, no ion produced by the elimination of the group linked to C-4 was detected [54,56]. 

#### 3.1.4. Bicyclic Sesterterpene Lactones Containing C-23 Carboxylic Acid

**Figure 8:**
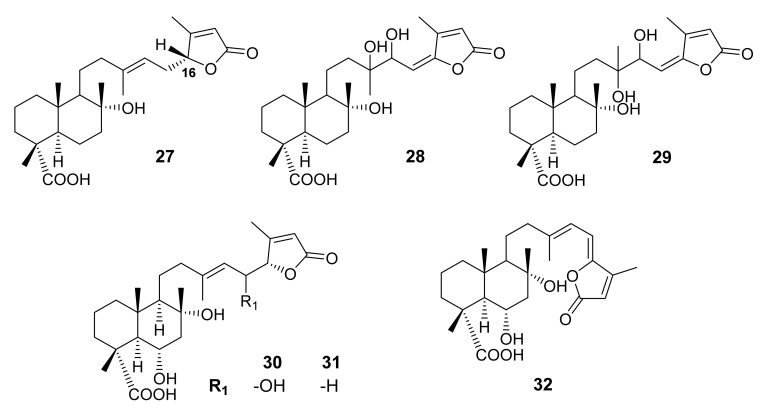
Structures of **27**–**32**.

Salvimirzacolide (**27**) (Figure 8), a bicyclic sesterterpene lactone whose C-23 possesses a carboxylic acid function, was isolated from the MeOH-soluble portion of an acetone extract of the aerial parts of *S. mirzayanii* Rech. and Esfandieri, collected from Malek and Adori villages near Kerman, Iran. Extensive NMR spectral analysis, in combination with X-ray analysis, revealed that **27** is a normal bicyclic sesterterpene (with 10*R* configuration) containing a γ-butyrolactone ring with the *R*-configuration at C-16 [57].

The sesterterpene lactones 8α,13,14-*threo*-trihydroxy-labd-15,17-dien-16,19-olide-23-oic acid (**28**) and 8α,13,14-*erythro*-trihydroxy-labd-15,17-dien-16,19-olide-23-oic acid (**29**) (Figure 8) were also isolated from the acetone extract of the aerial parts of *S. palaestina* Bentham, collected in Jordan. The NMR data of **28** and **29** were very similar except for the signals of H-12, H-14, C-13, C-14, C-21 and Me-21, suggesting that they are epimers at C-13/C-14. The relative configurations of C-13/C-14 *vic*-diol of **28** and **29** were established as *threo* and *erythro*, respectively, by observing the methyl signals of their acetonides in the ^1^H NMR spectra [53].

The sesterterpene lactones 6α,8α,15(*S*)-trihydroxy-23-carboxy-labd-13(14),17-dien-16*S*,19-olide (**30**), 6α,8α-dihydroxy-23-carboxy-labd-13(14),17-dien-16,19-olide (**31**), 6α,8α-dihydroxy-23-carboxy-labd-13(14),15,17-trien-16,19-olide (**32**) (Figure 8) were isolated from the CHCl_3_-MeOH fraction of the aerial parts of *S. dominica*. The structures of **30**–**32** were established by analysis of ^1^H and ^13^C NMR spectra and ESIMS data [54]. 

#### 3.1.5. Bicyclic Sesterterpene Lactones Containing C-23 Methyl Ester of Carboxylic Acid

**Figure 9:**
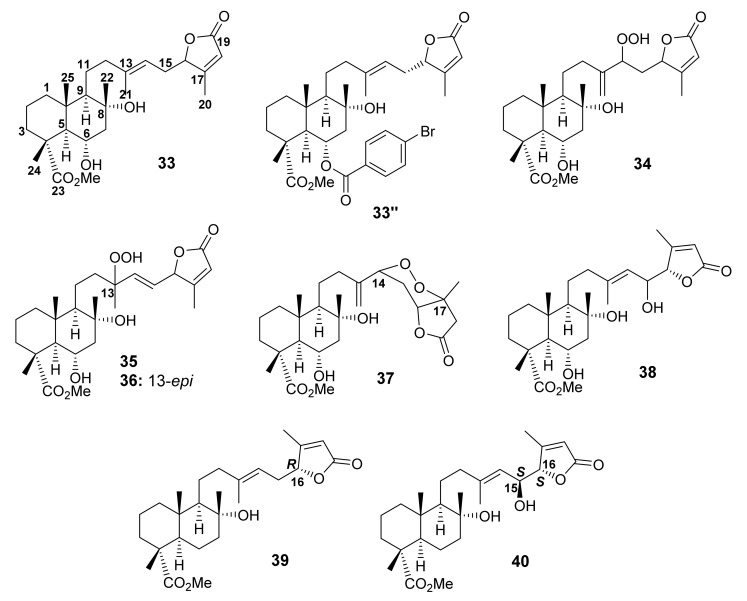
Structures of **33**–**40**.

In 1982, Rustaiyan et al. first described the isolation of a bicyclic sesterterpene lactone with a methyl ester of carboxylic acid at C-23 and named it salvileucolide methyl ester (**33**) (Figure 9) from a diethyl ether extract of the aerial parts of *S. hypoleuca* Benth, collected from the north of Teheran, Iran [58]. Later, Rustaiyan and Sadjadi also reported the isolation of **33** (Figure 6) from a MeOH-soluble fraction of *S. syriaca* L., collected from north of Taleghan, Iran [55]. Moghaddam et al. also reported the isolation of **33** from the aerial parts of *S. sahendica* Boiss & Buhse, an endemic plant of Iran which was collected in Bostanabad, East of Tabriz. However, neither the relative nor absolute configuration of C-16 in **33** was determined [59]. Only in 1996 did Linden et al. [60] succeed in determining the absolute configuration of the stereogenic carbons in **33** by performing an X-ray analysis of 6-*O*-*p*-bromobenzoyl ester of **33** (**33″**; Figure 9), whose ORTEP diagram putatively indicated the *R*-configuration at C-10 and C-16 [60]. Furthermore, salvileucolide methyl ester (SME) derivatives, *viz.* 14-hydroperoxy-13(21)-dehydro-SME (**34**), 13-hydroperoxy-14-ene-SME (**35**), 13-*epi*-hydroperoxy-14-ene-SME (**36**), and 14,17-cycloperoxy-13(21)-dehydro-SME (**37**) (Figure 9), were also isolated from the polar fraction of the aerial parts of *S. hypoleuca*. The structures of **34**–**37** were established by NMR spectral analysis [61].

Further bicyclic sesterterpene lactones containing C-23 carboxylic acid methyl ester such as 6α,8α,15(*S*)-trihydroxy-23-carboxymethyl-labd-13(14),17-dien-16*S*,19-olide (**38**) were isolated from *S. dominica* L. [54], while (4*R*,5*R*,8*R*,9*R*,10*S*,16*R*,13*E*)-8-hydroxy-23-carboxymethyl-labd-13(14),17(18)-dien-16,19-olide (**39**) and (4*R*,5*R*,6*S*,8*R*,9*R*,10*S*,15*S*,16*S*,13*E*)-8,15-dihydroxy-23-carboxymethyl-labd-13(14),17(18)-dien-16,19-olide (**40**) (Figure 9) were isolated from *S. tingitana* Etl. That the relative configuration of **39** and **40** was the same as that of **33** was based on the values of coupling constants of H-5 (*J* = 12.0 and 2.3 Hz) as well as NOESY correlations between H-5 and H-9, and between H_3_-22, H_3_-24, and H_3_-25 of **39** and **40,** which were consistent with a β-orientation of Me-22, Me-24, and Me-25 as well as a *trans* junction of the decalin ring system. Additionally, the absolute configuration of C-15 was determined as *S* by the Mosher’s method. Moreover, the vibrational circular dichroism (VCD) spectra of **39** and **40** were recorded and compared to their calculated spectra at the B3LYP/6-31+G(d,p) level of theory. It was found that the experimental and calculated VCD spectra showed a significantly better fit for the (4*R*, 5*R*, 8*R*, 9*R*, 10*S*, 16*R*)-**39**. On the other hand, the VCD similarity analysis gave no clear preference to any of the four calculated spectra for **40** [52].

#### 3.1.6. Bicyclic Sesterterpenes Containing C-6, 23- and C-16, 19-Diolide

**Figure 10:**
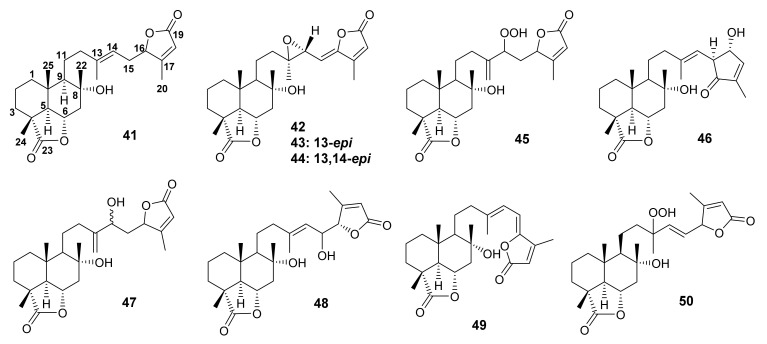
Structures of **41**–**50**.

Salvileucolide-6,23-lactone (**41**) (Figure 10), the first 6,23-lactone-containing sesterterpene reported from *Salvia* plants, was isolated from the diethyl ether extract of the aerial parts of *S. hypoleuca* Benth. Like **33**, the structure of **41** and the stereochemistry at C-5 and C-6 were established based on the observation of the ^1^H NMR spectrum through spin decoupling and the addition of the chemical shift reagent, Eu(fod)_3_, and the ^13^C NMR spectrum. However, the relative and absolute configurations of C-16 were not determined [58].

**Figure 11:**
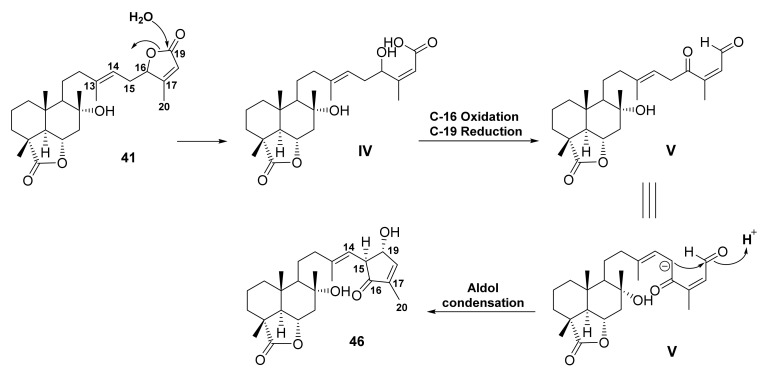
Proposed mechanism for the formation of 4-hydroxycyclopent-2-en-1-one in **46**.

The polar fraction of the aerial parts of *S. hypoleuca* also furnished 15,16-dehydrosalvileucolide-6,23-lactone-*trans*-epoxide (**42**), 15,16-dehydrosalvileucolide-6,23-lactone-*cis*-epoxide (**43**), and 15,16-dehydrosalvileucolide-6,23-lactone-13,14-bis*-epi-trans*-epoxide (**44**), 14-hydroperoxy-13(21)-dehydro-13,14-dihydro-salvileucolide-6,23-lactone (**45**), and salvileucolide (**46**), a sesterterpene with a 4-hydroxycyclopent-2-en-1-one moiety (Figure 10). The structures of **42**–**46** were identified by detailed analysis of ^1^H NMR spectra using a double irradiation technique. The structure of the 4-hydroxycyclopent-2-en-1-one in **46** was postulated to derive from a hydrolysis of 19,16-lactone in **41** to give a γ-hydroxy carboxylic acid in **IV**. Oxidation at OH-16 to ketone and reduction of COOH-19 to aldehyde yield the intermediate **V**. Aldol condensation of the intermediate **V** led to a formation of the 4-hydroxycyclopent-2-en-1-one moiety in **46** (Figure 11) [61].

Lachnocalyxolide B (**47**) (Figure 10) was isolated from a defatted acetone extract of the aerial parts of *S. lachnocalyx* Hedge, collected from Eghlid in the Fars Province, Iran. The structure of **47** was established by 1D and 2D NMR spectral analysis and HR-ESIMS data. The relative configuration of **47** was established based on NOESY correlations from H-6β to H_3_-22, H_3_-24, H_3_-25, and H-7β, as well as from H-5α to H-9α. However, the relative configurations at C-14 and C-16 were not assigned because of high conformational flexibility and free rotation around C-14/C-15 and C-15/C-16 [62]. 

The CHCl_3_-MeOH extract of the aerial parts of *S. dominica* L. also furnished 8α,15(*S*)-dihydroxy-labd-13(14),17-dien-23,6α-16*S*,19-diolide (**48**) and 8α-hydroxy-labd-13(14),15,17-trien-6α,23-16,19-diolide (**49**) (Figure 10). The structures of **48** and **49** were established by analysis of ESIMS and ^1^H and ^13^C NMR spectra. In the case the of **48**, the ROESY spectrum displayed correlations between Me-22, Me-24, and Me-25, thus confirming the α-orientation of hydroxyl group on C-8 [54].

A further sesterterpene diolide, 8α-hydroxy-13-hydroperoxylabd-14,17-dien-19,16;23,26α-diolide (**50**) (Figure 10), was isolated from the acetone extract of the aerial parts of *S. sahendica*, collected between Tabriz and Bostanabad, East Azerbaijan Province, Iran. The structure of **50** was elucidated by detailed analysis of 1D and 2D NMR experiments and HRESIMS. The relative configuration of the lactonized C-6 was determined as α, based on the coupling constant of H-6 with H-5 (*J*_5,6_ = 11 Hz) as well as by NOESY correlations from H-6 to H_3_-22, H_3_-24 and H_3_-25, all of which are β-oriented. Moreover, H-5 and H-9 also showed a NOESY correlation, implying a β-orientation of the side chain on C-9. However, the configurations of C-13 and C-16 were not determined [63].

#### 3.1.7. Bicyclic Sesterterpenes Containing C-19,16-Olide and C-6,23-Tetrahydrofuran

**Figure 12:**
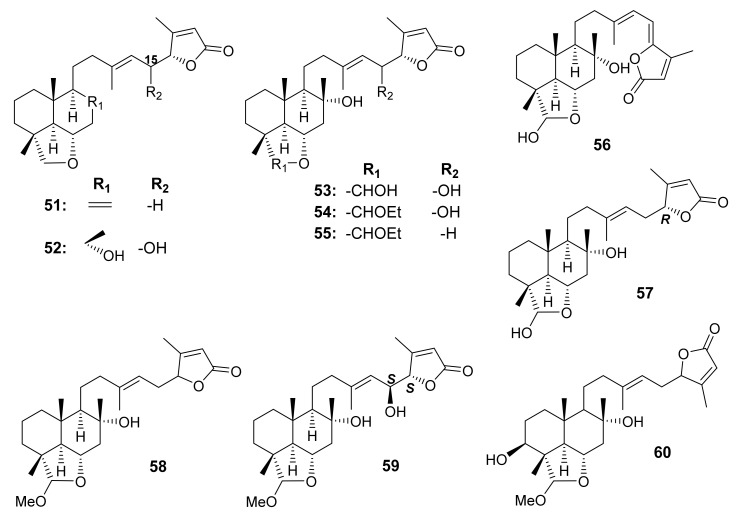
Structures of **51**–**60**.

The CHCl_3_-MeOH extract of the aerial parts of *S. dominica* L. also furnished 23,6α-epoxy-labd-8,13(14),17-trien-16*R*,19-olide (**51**), 15(*S*)-dihydroxy-23,6α-epoxy-labd-13(14),17-dien-16*S*,19-olide (**52**), 8α,15(*S*),23α-trihydroxy-23,6α-epoxy-labd-13(14),17-dien-16*S*,19-olide (**53**), 8α,15(*S*)-dihydroxy-23α-*O*-ethyl-23,6α-epoxy-labd-13(14),17-dien-16*S*,19-olide (**54**), 8α-hydroxy-23α-*O*-ethyl-23,6α-epoxy-labd-13(14),17-dien-16*R*,19-olide (**55**), and 8α,23-dihydroxy-23,6α-epoxy-labd-13(14),15,17-trien-16,19-diolide (**56**) (Figure 12). The structures of **51**–**56** were elucidated by analysis of 1D and 2D NMR spectra and HR-ESIMS data. The relative configuration of **51** was established by ROESY correlations, while the absolute configuration at C-15 of **52** was determined by a modified Mosher’s method. The decalin ring junction and the stereochemistry of the side chains of **51**–**56** were identical to those of **14** [54].

The *n*-hexane-insoluble portion of the CH_2_Cl_2_ extract of the fresh aerial parts of *S. tingitana* also furnished (4*R*,5*R*,6*S*,8*R*,9*R*,10*S*,16*R*,23*S*,13*E*)-8,23-dihydroxy-23,6-epoxy-labd-13(14),17(18)-dien-16,19-olide (**57**), (13*E*)-8α-hydroxy-23α-*O*-methyl-23,6α-epoxy-labd-13(14),17(18)-dien-16,19-olide (**58**), (4*R*,5*R*,6*S*,8*R*,9*R*,10*S*,15*S*,16*S*,23*S*,13*E*)-8,15-dihydroxy-23-*O*-methyl-23,6-epoxy-labd-13(14),17(18)-dien-16,19-olide (**59**) (Figure 12). The relative configurations at C-4, C-5, C-6, C-9, and C-10 of **57**, **58** and **59** were established based on the coupling constants of H-5, H-6, and H-9 as well as by NOESY correlations from H-6 to H_3_-22, H_3_-24, and H_3_-25, and from H-5 to H-9. By using a modified Mosher’s method, the configuration of C-15 in **59** was assigned as 15*S*. Tentative assignment of the absolute configurations of C-15 and C-16 by comparison of the calculated and experimental VCD spectra was unsuccessful since the results were in conflict with that obtained from the Mosher’s method. Therefore, the absolute configuration of C-16 in **57** and **59** was assigned by comparison of the NMR data with those reported for the congeners [52].

Hasan et al. isolated salvidominicolide B (**60**) (Figure 12) from the aqueous MeOH portion of the CHCl_3_ extract of whole parts of *S. dominica* L., which was collected in Al-Mastaba region, Jordan. The structure of **60** was established by detailed analysis of 1D and 2D NMR spectra and HRMS data [64].

### 3.2. Tricyclic Sesterterpenoids

**Figure 13:**
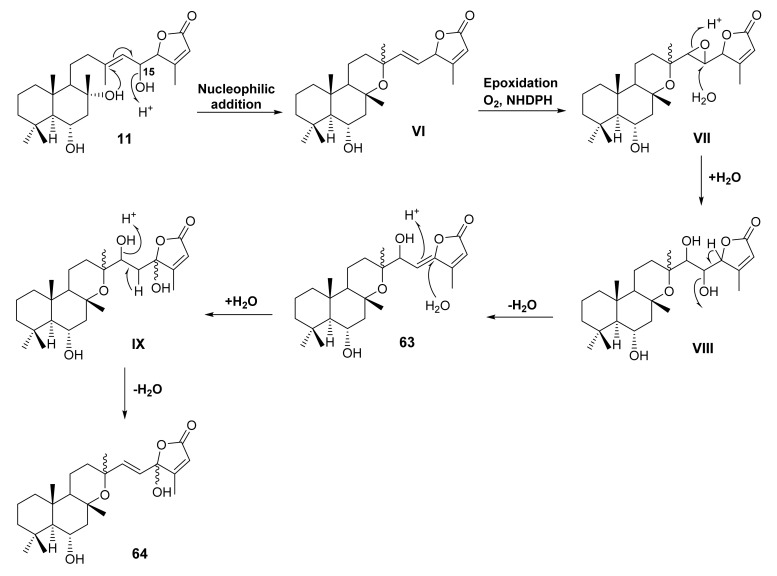
Proposed biosynthetic pathway for the formation of tricyclic sesterterpene lactones.

So far, 21 tricyclic sesterterpenoids containing a tetrahydropyran ring, angularly fused with a decalin ring system, have been reported from six *Salvia* species *viz. S. aethiopis*, *S. dominica*, *S. yosgadensis*, *S. tingitana*, *S. lachnocalyx*, and *S. mirzayanii*. Like bicyclic sesterterpenoids, this group of sesterterpenoids is categorized as C-23 methyl, C-23 carboxylic acid, C-23 carboxylic acid methyl ester, C-23,6 and C-16,19 diolides. The biosynthetic pathway of these compounds could originate from 8, 16-dihydroxy bicyclic sesterterpene containing γ-lactone, **11**, followed by nucleophilic addition of OH-8 to C-13 of the double bond with concomitant elimination OH-15 of the side chain to give **VI**. Epoxidation of **VI** gives **VII**, which upon hydroxylation of the C-14/C-15-epoxide gives **VIII**. Elimination of OH-15 by dehydration leads to a formation of **63**. Nucleophilic addition of the double bond at C-16 by H_2_O would give **IX,** which after dehydration generates a double bond between C-14 and 15 and a hemiacetal function at C-16 in **64** (Figure 13).

**Figure 14:**
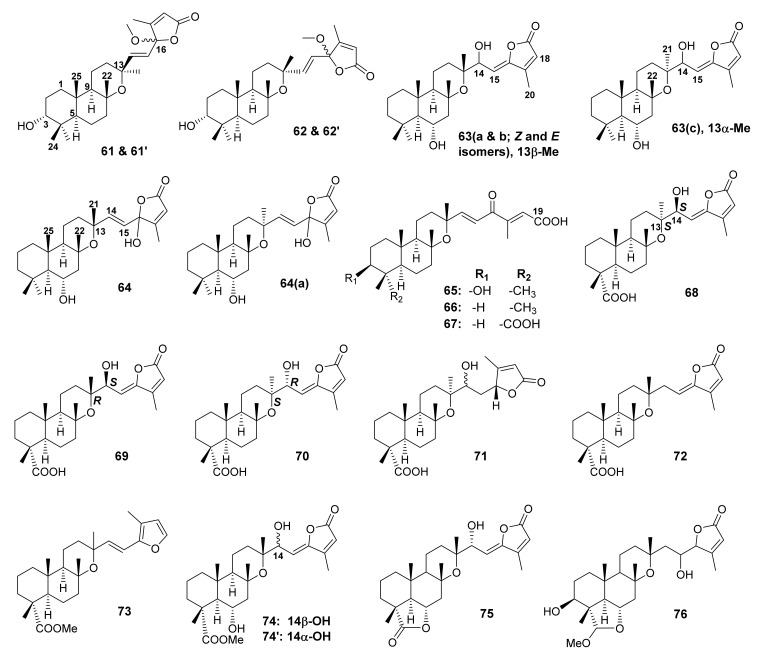
Structures of **61**–**76**.

Two γ-methoxybutenolide-containing sesterterpenes, 13-*epi*-salviaethiopisolide (**61** and **61′**) together with salviaethiopisolide (**62** and **62′**) (Figure 14), were isolated from the MeOH extract of the aerial parts of *S. aethiopis*, collected in Spain. The ^1^H and ^13^C NMR spectra of both compounds displayed a duplicity of several signals, with a relative intensity of 55/45, suggesting that the compounds were a mixture of two epimers. The structures of both compounds were elucidated by 1D and 2D NMR spectral analysis as well as chemical transformation. Analysis of the NMR data of the acetylation and reduction products confirmed that both compounds were epimeric at C-16. The relative stereochemistry of **61/61′** and **62/62′** was determined by decoupling constants of each proton and the results of the NOE experiments that showed clear effects for H-25 with H-24, H-2 and H-1, H-24 with H-23, H-3 with H-2, H-24 and H-23, and H-5 with H-9 and H-1, suggesting the β-orientation for Me-22, Me-24 and Me-25, and the α-orientation for H-5 and H-9 [65]. 

Yosgadensolide A (6α,14-dihydroxymanoyloxide-15,17-dien-16,19-olide; **63**) and yosgadensolide B (6α,16-dihydroxymanoyloxide-14,17-dien-16,19-olide; **64**) (Figure 14) were isolated from the acetone extract of the aerial parts of *S. yosgadensis* Freyn et Bornm., collected in Turkey. In the case of **63**, the ^1^H and ^13^C NMR spectra of its two acetylation products were very similar except for the signals of C-14, C-15, C-18, and C-20, which were slightly different, indicating the possible presence of *E*- and Z-stereoisomers (**a** & **b**). Thus, the structures of **63a** and **63b** were established as 6α,14-dihydroxymanoyloxide-15,17-dien-15(*Z*)-16,19-olide and 6α,14-dihydroxymanoyloxide-15,17-dien-15(*E*)-16,19-olide, respectively (Figure 14). The ^1^H and ^13^C NMR spectra of another co-isolated compound (**63c**) resembled those of **63** except for the signals of H-14, H-15, Me-21 and Me-22, indicating its gross structure was similar to that of **63a**/**63b**. The authors suggested that **63c** was a C-13 epimer of **63**; however, due to the limited amount of the compound, its stereochemistry was not investigated. In turn, the ^1^H and ^13^C NMR spectra of another co-isolated sesterterpene (**64**) were similar to those of **61** and **61′**, indicating the presence of a sesterterpene structure derived from manoyloxide, but with different side-chain from that of **61**. However, careful analysis of the ^1^H and ^13^C NMR data of **64** revealed its similarity both in the side chain and in the main skeleton to those of **62/62′**. The obvious difference is the presence of a hydroxyl group on C-16 in **64** instead of a methoxy group in **62/62′** and a secondary hydroxyl group on C-6 in **64** instead of on C-3 in **62/62′**. Therefore, **64** was identified as 6α,16-dihydroxy-manoyloxide-14,17-dien-16,19-olide (Figure 14). The ^1^H and ^13^C NMR data of another co-isolated sesterterpene lactone (**64a**) resembled those of **64** except for the chemical shift values of H-14, H-15, Me-21, Me-22, and Me-25, suggesting that **64a** could be a C-13 epimer of **64** and this hypothesis was corroborated by NOE experiment. Therefore, the structure of **64a** was elucidated as 6α,16-dihydroxy-13-*epi*-manoyloxide-14,17-dien-16,19-olide (Figure 14) [66].

Three tricyclic sesterterpenes, *viz.* 3β-hydroxymanoyloxide-14(*E*),17-dien-16-oxo-19-oic acid (**65**), hydroxymanoyloxide-14,17-dien-16-oxo-19-oic acid (**66**), and hydroxymanoyloxide-14,17-dien-16-oxo-19,23-dioic acid (**67**) (Figure 14) were isolated from the CHCl_3_ extract of the aerial part of *S. aethiopis* L., obtained from plants cultivated in a greenhouse at Instituto di Genetica Vegetale, CNR, Portici, Napoli, Italy. The structures of **65**–**67** were established by 1D and 2D NMR spectral analysis and HR-ESIMS data. The relative stereochemistry at C-5, C-8, C-9, C-10, and C-13 was established by the values of coupling constant of H-5 and H-9 while their axial orientation was determined by observation of cross peaks from H-9 to H-5, Me-25, Me-21 to Me-22 in the NOESY spectrum, thus indicating a *trans-anti-trans* tricyclic moiety [67].

The manoyloxide-type sesterterpenes, **68**–**72** (Figure 14) were isolated from the acetone extract of the aerial parts of *S. mirzayanii*, an endemic species to Iran, which was collected at Geno Mountain in the Bandar Abbas, South of Iran. The structures of the compounds were elucidated by 1D and 2D NMR spectral analysis and HRESIMS data. The relative configurations of the compounds were established by NOESY correlations, while the absolute configurations at C-13 and C-14 of **68**, **69** and **70** were established by comparison of their calculated and experimental electronic circular dichroism (ECD) spectra. Therefore, the structures of **68**, **69** and **70** were elucidated as (4*R*,5*R*,8*R*,9*R*,10*S*,13*S*,14*S*)-14-hydroxymanoyloxide-15,17-dien-15(*Z*)-16,19-olide, (4*R*,5*R*,8*R*,9*R*,10*S*,13*R*,14*S*)-14-hydroxymanoyloxide-15,17-dien-15(*Z*)-16,19-olide, and (4*R*,5*R*,8*R*,9*R*,10*S*,13*S*,14*R*)-14-hydroxymanoyloxide-15,17-dien-15(*Z*)-16,19-olide, respectively. On the other hand, due to high conformational flexibility and free rotation around C-14/C-15 in **71**, the absolute configuration of C-14 could not be determined. Consequently, the structure of **71** was established as (4*R*, 5*R*, 8*R*, 9*R*, 10*S*, 13*S*, 14*R*)-14-hydroxymanoyloxide-15,17-dien-15(*Z*)-16,19-olide. For **72**, the ECD spectrum showed a positive Cotton effect (CE) at 290 nm (n → π* transition) and a negative CE at 235 nm (π → π* transition of α, β-unsaturated-γ-butenolide). The calculated ECD spectrum of the (4*R*, 5*R*, 8*R*, 9*R*, 10*S*, 13*R*)-stereoisomer showed a strong positive CE at 290 nm that fitted well with the experimental data. However, the negative CE at 230 nm in the experimental spectrum was absent in the calculated spectrum. Therefore, the ECD spectrum for the (4*R*, 5*R*, 8*R*, 9*R*, *10S*, 13*S*)-stereoisomer was calculated but showed a negative CE at 290 nm. Therefore, the optical rotations for both stereoisomers were also calculated and compared with the experimental data to confirm the absolute configuration, thus establishing the structure of **72** as (4*R*,5*R*,8*R*,9*R*,10*S*,13*R*)-manoyloxide-15,17-dien-15(*Z*)-16,19-olide [68].

A manoyloxide-type sesterterpene with a furan-containing side chain and a carboxylic acid ester function at C-23, (14*E*)-methylmanoyloside-14,16,18-trien-16,19-oxide-23-carboxilate (**73**) (Figure 14), was also isolated from the n-hexane-insoluble fraction of the CH_2_Cl_2_ extract of the aerial parts of *S. tingitana*. The structure of **73** was elucidated by extensive analysis of 1D and 2D NMR spectra and HR-ESIMS data. The NOESY correlations from H_3_-22 to H_3_-24, H_3_-25 and from H-5 to H-9 indicated that the relative configuration of **73** was the same as that of the previously reported manoyloxide-type sesterterpenoids. However, the relative configuration of C-13 remained unassigned due to overlapping signals of H_3_-21 and H_3_-22 [52].

Lachnocalyxolides A (**74**) and C (**75**) (Figure 14) were isolated from the ethyl acetate (EtOAc)-soluble fraction of the acetone extract of the aerial parts of *S. lachnocalyx* Hedge, collected in Iran. Like some previously discussed manoyloxide-type sesterterpenes, the ^1^H NMR spectrum of **74** showed two pairs of signals of H-14 and H-15 while the ^13^C NMR spectrum exhibited two pairs of the carbon signals of the two double bonds (C-15/C-16 and C-17/C-18) and one pair of a carbinol proton (C-14), indicating the presence of an epimeric pair (**74** and **74′**; Figure 14). Compound **75** was elucidated as a manoyloxide-type sesterterpene 6,23-olide with a hydroxyl group on C-14 and an α,β-unsaturated-γ-lactone in the side chain by ^1^H and ^13^C NMR data. The relative configuration of **75** was corroborated by NOESY correlations from H-6β to H_3_-22, H_3_-24, H_3_-25, and H-7β, as well as from H_3_-22 to H_3_-21, confirming that they are cofacial. Since diagnostic NOESY correlations were observed from H-14 to H_3_-21, H-12β, and H-12α, the predominant conformation of **75** is the one having gauche interactions of H-14 with both C-21 and C-12, indicating the configuration of the hydroxyl group on C-14 [62].

Hasan et al. reported the isolations of salvidominicolide A (**76**), a manoyloxide-type sesterterpene with 6,23-pyran moiety and a side chain containing α, β-unsaturated-γ-lactone (Figure 14) from *S. dominica* L. The structure of **76** was elucidated by 1D and 2D NMR spectral analysis and HRMS data. However, the stereochemistry of C-15 was not determined [64].

### 3.3. Norsesterterpenes

**Figure 15:**
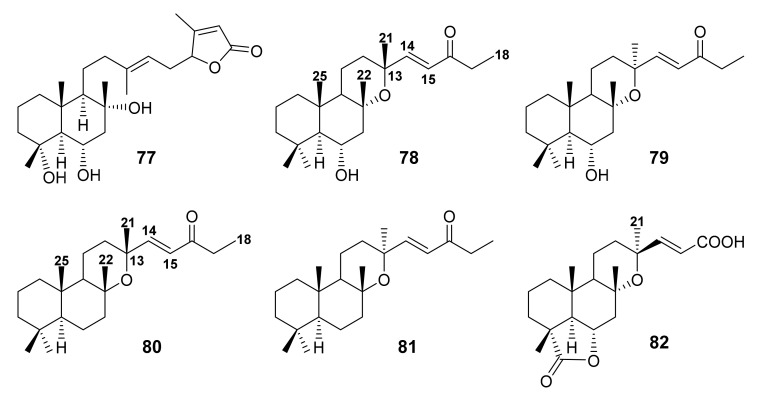
Structures of **77**–**82**.

The *n*-hexane-insoluble portion of the CH_2_Cl_2_ extract of the aerial parts of *S. tingitana* also furnished a C-23 norsesterterpene (**77**) (Figure 15) whose planar structure was established by 1D and 2D NMR spectral analysis and HRMS data. The NOESY correlations from H-6 to H_3_-22, H_3_-24, and H_3_-25, and from H-5 to H-9 indicated a *trans*-junction of the decalin ring system and a β-orientation of H-6 and Me-24. However, the stereochemistry of C-16 of the lactone ring was not determined. Consequently, the structure of **77** was established as (13*E*)-4α,6α,8α-trihydroxy-labd-13(14),17(18)-dien-16,19-olide. It is noteworthy mentioning that **77** is the first C-23 norsesterterpene from a *Salvia* species [52]. 

In a continuing investigation of Turkish *Salvia* species, Topcu et al. reported the isolation of dinorsesterterpenes, yosgadensonol (**78**) and 13-*epi*-yosgadensonol (**79**) (Figure 15) from the acetone extract of the aerial parts of *S. yosgadensis*, collected from Central Turkey (near Sultanhani, Konya). The structures of **78** and **79** were elucidated by HREIMS, 1D and 2D-NMR spectral analysis. The 1D and 2D NMR spectra and the molecular formula of both compounds (C_23_H_38_O_3_) identified them as 19,20-dinorsesterterpenes possessing the same tricyclic ring system as those of manoyloxide-type sesterterpenes but differ in the stereochemistry of C-13. The stereochemistry of C-16 in both compounds was determined by observing the NOE effects of Me-21 and Me-25 signals upon irradiation of Me-22 [69]. 

Another two dinorsesterterpenes, 6-dehydroxy-yosgadensonol (**80**) and 6-dehydroxy-13-*epi*-yosgadensonol (**81**) (Figure 15), were reported form the acetone extract of the aerial parts of *S. limbata* C. A. Meyer. The structures of the compounds were elucidated by interpretation of the ^1^H and ^13^C NMR spectra and comparison of their NMR data with those of **78**. The structure of **81** was established based on the slightly different proton chemical shift values of Me-21, Me-22 and Me-25 [70].

A C-17, C-18, C-19, and C-20 tetranorsesterterpene, (17,18,19,20-tetranor-13-*epi*-manoyloxide-14-en-16-oic acid-23,6α-olide; **82**) (Figure 15), was also isolated from the acetone extract of the aerial parts of *S. sahendica*. The structure of **82** was elucidated by HRESIMS and 1D and 2D NMR spectral analysis. The relative configuration at C-6 of the 23,6-lactone was determined as *R* on the basis of the magnitude of the coupling constants of H-6 as well as NOESY correlations from H-6 to H_3_-22, H_3_-24, and H_3_-25, all β-oriented. On the other hand, Me-21 was established as α-oriented due to a lack of NOE enhancement upon irradiation of Me-22. It is important to note that **82** is the first tetranorsesterterpene to have been reported from the genus *Salvia* [63].

## 4. Dammarane Triterpenoids

**Figure 16:**
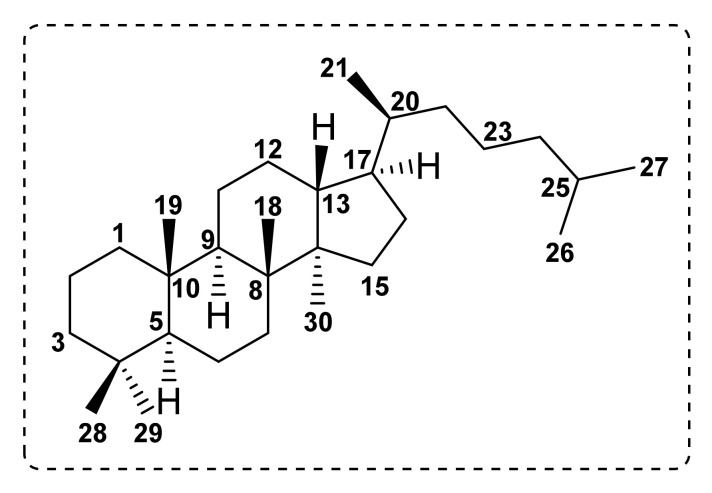
Basic carbon skeleton of dammarane-type triterpenoids.

Dammarane-type triterpenoids are tetracyclic triterpenoids whose structural feature is characterized by a 6/6/6/5 ring system with H-5α, H-9α, H-13β, three β-CH_3_ groups on C-4 (CH_3_-28), C-8 (CH_3_-20), C-10 (CH_3_-19), one α-CH_3_ group on C-14 (CH_3_-30), C-17β-side chain, and 20*R* or *S* configuration (Figure 16) [71]. These compounds are widely distributed in various plant families such as Araliaceae (*Aralia* sp., *Panax* sp., *Polyscias* sp.), Meliaceae (*Aglaia* sp.), Scrophulariaceae (*Bacopa* sp.), Celastraceae (*Celastrus* sp.), Rhizophoraceae (*Ceriops* sp.), Arecaceae (*Copernicia* sp.), Oleaceae (*Forsythia* sp.), Cucurbitaceae (*Gynostemma* sp.), Anacardidaceae (*Kageneckia* sp., *Myrica* sp., *Rhus* sp.) and Lamiaceae (*Salvia* sp.) [72].

**Figure 17:**
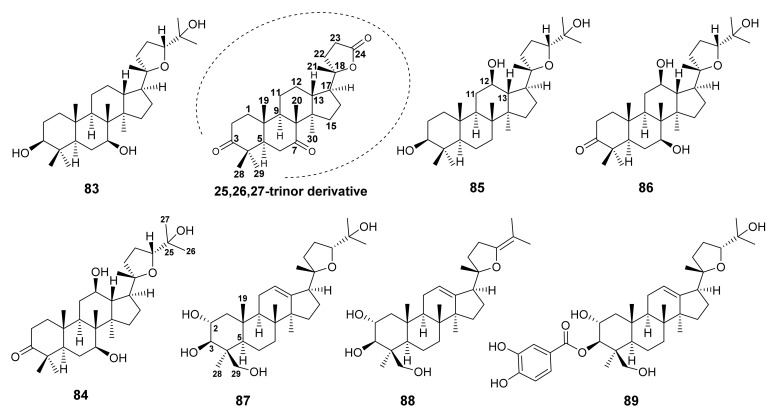
Structures of **83**–**89**.

Salvilymitol [(20*S*, 24*R*)-epoxydammarane-3β,7α,25-triol] (**83**) and salvilymitone (7β,25-dihydroxy-(20*S*,24*R*)-epoxydammaran-3-one) (**84**) (Figure 17) were isolated from the acetone extract of the aerial parts of *S. hierosolymitana* Boiss., which was collected from the botanic garden of Palermo, Italy. The structures of both compounds were elucidated by electron impact mass spectrometry (EIMS) and analysis of ^1^H and ^13^C NMR spectra. By analysis of the ^13^C NMR data, it was impossible to deduce the absolute configurations of the stereogenic carbons of the pyran ring in the side chain of **84** as (20*S*, 24*R*) or (20*R*, 24*S*). However, the configurations of these carbons were conclusively determined as (20*S*, 24*R*) by a single-crystal X-ray crystallography of the 25, 26, 27-trinor-γ-lactone derivative, a degradation products of **84** with Jones’ reagent [73]. 

Kolak et al. [74] reported the isolation of pixynol [(20*S*,24*R*)-epoxydammarane-3β,12β,25-triol] (**85**) (Figure 17) from the acetone extract of roots of *S. barrelieri* Ettling, an endemic *Salvia* species to Algeria collected from Ammoucha-Setif district in Northeastern Algeria. Although **85** was first reported from a lichen *Pyxine endochrysina* NYL. [75], and later from the acetone extract of roots of *S. bicolor*, collected in Malaga, Spain [76], the absolute configurations of C-20 and C-24 had not been determined. However, Kolak et al. [74] were able to obtain a suitable crystal of **85** for X-ray analysis and have assigned a complete stereochemistry of **85**.

In addition to **85**, Valverde et al. also isolated (20*S*, 24*R*)-epoxydammar-12β, 25-diol-3-one (**86**) (Figure 17) from the acetone extract of roots of *S. bicolor*. The β-orientation of the hydroxyl group on C-12 was proposed by two large axial-axial coupling constants (*ca.* 10.4 Hz) of H-12 with H-13 and H-11β while the absolute configurations of C-20 and C-24 were assigned by ^13^C chemical shift values as well as comparison of the ^13^C chemical shift values with those of the model compound [76]. 

The EtOAc-soluble fraction of the MeOH extract of the whole plant of *S. santolinifolia* Boiss., collected in Karachi, Pakistan, furnished three epoxydammarane triterpenes, santolins A-C (**87**–**89**) (Figure 17). The relative configurations at C-2, C-3, and C-4 of **87** were confirmed by the NOESY correlations from Me-28α to H-3 and H-5, as well as from Me-19β to H-2, and H-29. Thus, the structures of **87**–**89** were assigned as (2α,3β,20*S*,24*R*)-20,24-epoxydammar-12-ene-2,3,25,29-tetraol, (2α,3β,20*S*)-20,24-epoxydammar-12,24-dien-2,3,29-triol, and (2α,3β,20*S*,24*R*)-20,24-epoxy-2,25,29-trihydroxydammar-12-ene-3-yl 3,4-hydroxybenzoate, respectively [77]. 

**Figure 18:**
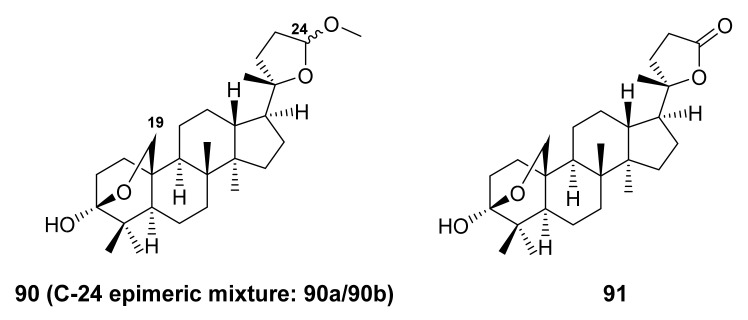
Structures of **90** and **91**.

Esquivel et al. [78] reported the isolation of the undescribed trinordammarane triterpene, amblyol (**90**) and the previously reported amblyone (**91**) [79] (Figure 18) from the acetone extract of the aerial parts of *S. aspera*, collected in the state of Plueba, Mexico. Compound **90** was isolated as a C-24 epimeric mixture as revealed by duplicate signals for most of the carbons in the ^13^C NMR spectrum. This hypothesis was corroborated by treatment of **90** with Jones reagent to give **91**, as well as acetylation of **90** with Ac_2_O/pyridine in the presence of 4-dimethylaminopyridine to give two OAc-19. Finally, the stereostructure of one epimer was obtained by single-crystal X-ray crystallography. 

**Figure 19:**
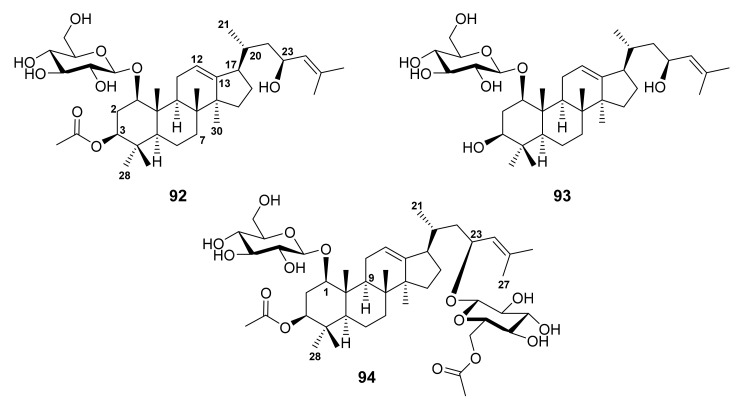
Structures of **92**–**94**.

Recently, Hafez Ghoran et al. described the isolation of undescribed darmmarane-type triterpenoid saponins containing glucose moieties, russelliinosides A-C (**92**–**94**) (Figure 19), from the CH_2_Cl_2_ extract of the aerial parts *S. Russellii* Beneth., collected in Northwestern Iran. The relative configurations of **92**–**94** were established by NOESY correlations. Therefore, the structure of **92**–**94** were elucidated as 1-*O*-[β-d-glucopyranosyl]-3β-*O*-acetoxy-23-hydroxydammar-12,24-diene, 1-*O*-[β-d-glucopyranosyl]-3β,23-dihydroxydammar-12,24-diene, and 1-*O*-[β-d-glucopyranosyl]-3β-*O*-acetoxy-23-[6-*O*-acetoxy-β-d-glucopyranosyl]-dammar-12,24-diene, respectively. In all compounds, the glucose motifs were identified by a total correlation spectroscopy (TOCSY) correlations. Compounds **92**–**94** are the first Δ^12,13^ and C-20 saturated dammarane saponins [80].

## 5. Triterpenoids with Novel Skeleton

**Figure 20:**
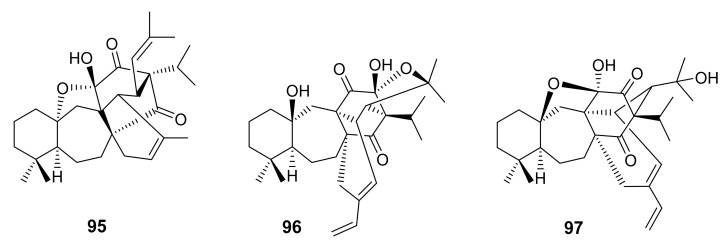
Structure of **95**–**97**.

**Figure 21:**
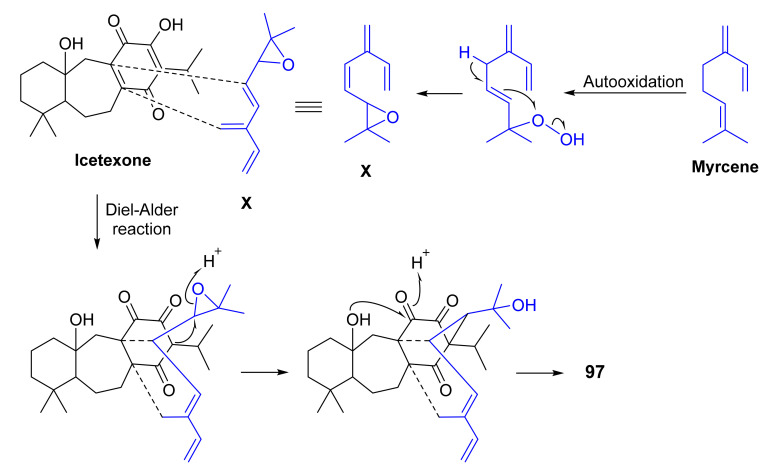
Proposed biogenesis of salvadiol (**97**) through Diel–Alder reaction of icetexone with autoxidation product of myrcene (**X**).

Ahmad et al. described the isolation of salvadiones A (**95**) and B (**96**), two C_30_-triterpenoids with a novel and rare carbon skeleton of five carbocycles, from the *n*-hexane soluble fraction of *S. bucharica* M. Pop, collected from Quetta, Baluchistan, Pakistan. The structures of **95** and **96** (Figure 20) were established by extensive analysis of 1D and 2D NMR spectra and were confirmed by single-crystal X-ray diffraction analysis [81]. Furthermore, the same research group has also reported the isolation of another triterpene of the same carbon skeleton, named salvadiol (**97**) (Figure 20) from the n-hexane soluble fraction of the same plant and whose structure was established by 1D and 2D NMR spectral analysis and single-crystal X-ray diffraction. The authors have proposed a biosynthetic pathway for **97** through a Diels-Alder type reaction of icetexone diterpene precursor with **X**, which was derived from an autoxidation of the monoterpene myrcene (Figure 21) [82].

**Figure 22:**
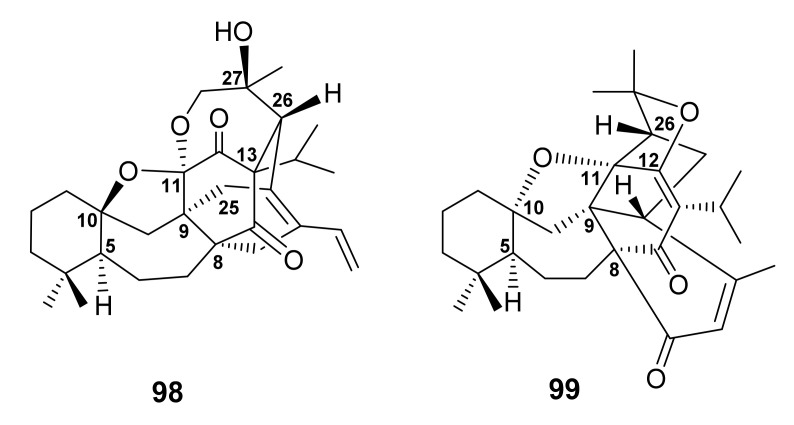
Structures of **98** and **99**.

**Figure 23:**
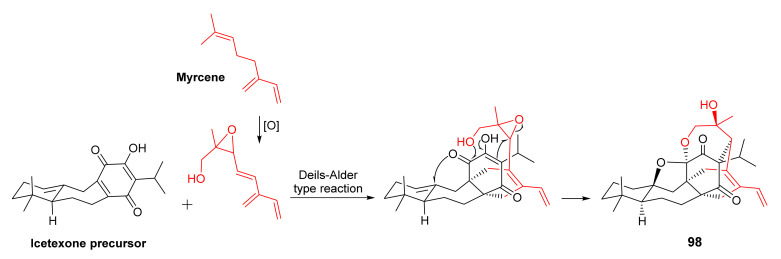
Proposed biosynthetic pathway for **98**.

The antiplasmodial *n*-hexane extract of the aerial parts of *S. hydrangea* DC. ex Benth., collected from the Koohin region in Qazvin Province, Iran, furnished salvadione C (**98**) and perovskone B (**99**) (Figure 22), two triterpenes of rare skeleton. The structures of **98** and **99** were elucidated by extensive analysis of 1D and 2D NMR spectra. The absolute configurations of the stereogenic carbons in **98** and **99** were established as 5*S*,8*R*,9*S*,10*S*,11*R*,13*R*,25*R*,26*R*,27*S* and 5*R*,8*R*,9*R*,10*R*,11*S*,12*R*,26*S*, respectively, by comparison of their experimental and calculated ECD spectra. Since the triterpene skeleton of **98** and **99** is the same as that of salvadiol (**97**), a similar biosynthetic route was proposed for **98** as shown in Figure 23 [83].

**Figure 24:**
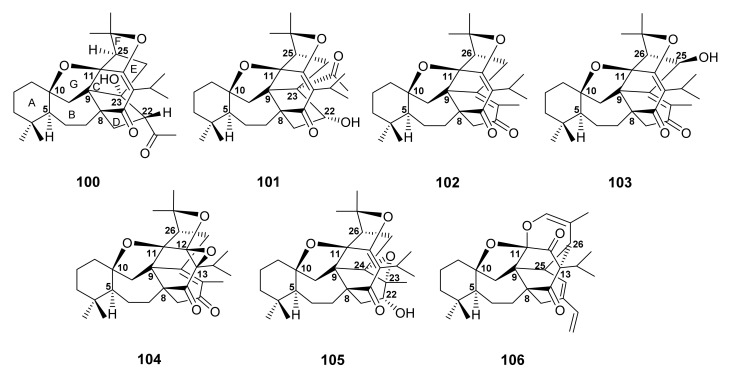
Structures of **100**–**106**.

Further examination of the *n*-hexane extract of the aerial parts of *S. hydrangea* by the same research group led to the isolation of hydrangenone (100) (Figure 24), another heptacyclic triterpenoid with a 6/7/6/5/5 ring system similar to that of **98** and **99**. The structure of **100** was elucidated by extensive analysis of 1D and 2D spectra. The relative configuration of **100** was established by NOESY correlations as well as by single-crystal X-ray analysis while the absolute structure was established as 5*S*,8*R*,9*R*,10*S*,11*R*,22*R*,23*R*,25*R* by comparison of the experimental and calculated ECD spectra [84].

**Figure 25:**
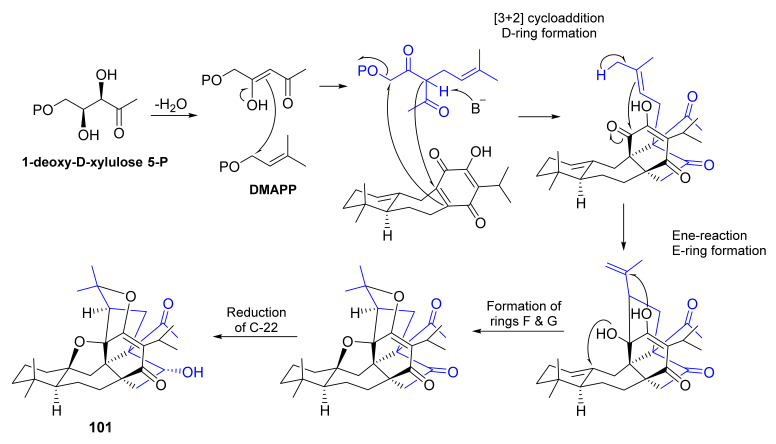
Proposed biosynthetic pathway for **101**.

Continuation of a phytochemical investigation of the n-hexane extract of the aerial parts of *S. hydrangea* allowed Tabefam et al. to isolate further six unreported triterpenoids with rare skeleton, named hydrangenone B (**101**), pervoskones C-F (**102**–**105**) and salvadione D (**106**) (Figure 24), in addition to **95** (Figure 20). The structures of the isolated compounds were elucidated by comprehensive analysis of 1D and 2D NMR spectra and HRMS data. The absolute structures of all the compounds were determined by comparison of the calculated and experimental ECD spectra. In the case of **95**, the absolute configurations of the stereogenic carbons were determined by single-crystal X-ray diffraction analysis using CuKα radiation. As a result, the absolute configurations of C-5, C-8, C-9, C-10, C-11, C-13, C-24 and C-25 are established as follows: **95** (5*S*,8*R*,9*S*,10*S*,11*R*,13*R*,24*R*,25*R*), **101** (5*S*,8*R*,9*R*,10*S*,11*R*,22*R*,23*S*,25*S*), **102** (5*S*,8*R*,9*S*,10*S*,1 1*R*,26*R*), **103** (5*S*,8*R*,9*S*,10*S*,11*R*,25*R*,26*R*), **104** (5*S*,8*R*,9*S*,10*S*,11*R*,12*R*,13*S*,26*R*), **105** (5*S*,8*R*,9*R*,10*S*,11*R*,22*R*,23*R*,24*S*,26*R*), and **106** (5*S*,8*R*,9*S*,10*S*,11*R*,13*R*,25*R*,26*S*). Additionally, a proposed biosynthetic pathway for **101** is shown in Figure 25 [85].

It is interesting to note that since this group of C_30_-terpenes is proposed to derive from the Diel–Alder reaction between icetexone-type diterpenoid and an autoxidation product of myrcene (a monoterpene), they are sometimes referred to as “isoprenoids” to distinguish them from normal triterpenoids, which are formed by direct cyclization of 2,3-oxidosqualene.

## 6. Biological and Pharmacological Activities of Uncommon Terpenoids from *Salvia* Species

### 6.1. Antioxidant Activity

As expected, a majority of terpenoid compounds do not possess relevant antioxidant activity. However, a few terpenoids from *Salvia* species were found to exhibit weak antioxidant properties. For example, *rel*-(5*S*,6*S*,7*S*,10*R*,12*S*,13*R*)-7-hydroxyapiana-8,14-diene-11,16-dion-(22,6)-olide (**4**) (Figure 2) exhibited a weak activity in both DPPH assay and the oil stability index (OSI) with methyl linoleate [86]. Pixynol (**84**)(Figure 17) also exhibited a weak antioxidant activity when compared to butylated hydroxytoluene (BHT) and α-tocopherol, in β-carotene-linoleic acid assay, followed by DPPH^•^, ABTS^•+^, and O_2_^•−^ radicals, with IC_50_ values > 100 μg/mL [75].

### 6.2. Antiviral Activity

Since some 1,3-diketone-containing secondary metabolites are known as HIV-1 integrase inhibitors [87], Xu et al. tested **7** and **8** (Figure 3) for their cytopathic effects against HIV-1. Compound **7** displayed better anti-HIV-1 activity than **8**, with half-maximal effective concentration (EC_50_) values of 40.74 and 89.13 μg/mL, respectively (selectivity index; SI of 2.19 and 1.78, respectively). Interestingly, it was found that the more carbonyl groups the compounds have the weaker anti-HIV-1 effects the compounds exhibit [48].

### 6.3. Cytotoxic Activity

The in vitro cytotoxic activity assay showed that **21**–**26** (Figure 7) were inactive against three cancer cell lines *viz.* Hela ((cervical cancer), HEK293 (human embryonic kidney), and J774.A1 (mouse monocyte macrophage) at concentrations higher than 100 μM [54,56].

A series of sesterterpenes, *viz.* **11** (Figure 5), **14**, **15** (Figure 6), **21**, **22** (Figure 7), **30**–**32** (Figure 8), **38** (Figure 9), **48** (Figure 10), **51**–**55**, **60** (Figure 12), **65**–**67**, **68**–**72**, and **76** (Figure 14) isolated from *S. dominica* were tested for inhibitory activity against TTL, a promising target for new anticancer therapeutics that is involved in the tyrosination cycle of the C-terminal of tubulin in cancer cells, by Surface Plasmon Resonances (SPR) studies to obtain the kinetic and thermodynamic parameters of the ligand-protein complex formation. Interestingly, 18 out of the 24 compounds examined effectively interacted with TTL. Compounds **15**, **51**, **65**, and **76** had the best pseudothermodynamic dissociation constants (KD) values of 9.3 × 10^−8^, 0.7 × 10^−8^, 7.2 × 10^−8^, and 7.3 × 10^−8^ M, respectively. On the other hand, **21**, **22**, **38**, and **66**, featuring one or two free hydroxyl groups at C-14 and C-15 in the side chain, were inactive. The presence of C-15/C-16 double bond, 23,6α-epoxy ring (in **38**, **68**, **69**, and **71**), and 23,6α-γ-lactone ring (in **30** and **70**) obviously decreased the affinity for the enzyme, but a C-13/C-14 double bond and C-15 methylene group were essential for the activity. Furthermore, treatment of MCF-7 (human breast cancer) cells with the most active compound, **31** (with KD of 4.7 × 10^−8^ M from SPR assays) at a concentration of 100 μM for 24 and 48 h and then analyzed by Western blot Δ-2 tubulin levels, showed that **31** significantly penetrated the membrane and inhibited TTL inside the cancer cell. Thus, **31** could be considered a good lead for further drug developments to design a better drug because of its 10-fold higher activity than other compounds [54,56].

Compounds **33** (Figure 9), **41** (Figure 10), **74**, and **75** (Figure 14), isolated from *S. lachnocalyx*, were evaluated against two human cancer-cell lines, HeLa and MCF-7, using the MTT ((3-(4,5-dimethylthiazol-2-yl)-2,5-diphenyltetrazolium bromide) assay. All sesterterpenes showed weak cytotoxicity, with IC_50_ values higher than 50 μM, when compared with paclitaxel (a positive control, IC_50_ values of 0.004 and 0.028 μM, respectively) [62].

The dammarane triterpenoid saponins **92**–**94** (Figure 19) were evaluated for their cytotoxicity against MCF-7 and A549 cell lines; however, only **92** and 93 exhibited cytotoxicity against MCF-7 (IC_50_ = 7.1 and 30.7 μg/mL, respectively) and A549 (IC_50_ = 33.9 and 69.4 μg/mL, respectively). Analysis of structural features of these saponins suggested that the acetoxy group on C-3 increased the cytotoxicity, while C-23 glycosylation decreased the cytotoxicity [80].

### 6.4. Antiparasitic Activity

Antiplasmodial activity-guided fractionation of *S. hydrangea* by n-hexane, EtOAc, and MeOH, revealed that the n-hexane fraction was active against *Plasmodium falciparum* K1 and *Trypanosoma brucei rhodesiense* STIB900, with IC_50_ values of 3.2 and 18 μg/mL, respectively. Among the isolated compounds from this active fraction, **98** and **99** (Figure 22) displayed potent antiplasmodial activity with IC_50_ values of 1.43 and 0.18 μM, respectively (SI of 86.2 and 69.6). On the other hand, **98** and **99** showed moderate potency against *T. brucei rhodesiense* STIB900 (IC_50_ values of 4.33 and 15.92 μM, respectively) [83]. On the contrary, **100** (Figure 23) displayed moderate in vitro activity against *P. falciparum* K1, with IC_50_ = 1.4 μM, SI = 6, cytotoxicity in rat myoblast (L6) cells. The IC_50_ of the positive control, artesunate, was 0.1 μM [84]. Compounds **95** (Figure 20), **101**–**106** (Figure 24) were assayed for their in vitro inhibitory activity against some protozoan parasites including *P. falciparum* (NF54), *T. brucei rhodesiense* (STIB900) trypomastigotes, *T. cruzi* (Tulahuen C4) amastigotes, and *Leishmania donovani* (MHOM-ET-67/L82) amastigotes. Curiously, *P. falciparum* was found to be the most sensitive parasite to the tested compounds, with IC_50_ values ranging from 0.6 μM (for **102**) to 7.9 μM (for **101**). On the other hand, none of the tested compounds showed selective toxicity toward *T. brucei rhodesiense* and *L. donovani* (SI ≤ 2.4 and SI ≤ 5.9, respectively). Moreover, **102** and **103** were found to be the most active inhibitors against *T. cruzi* parasite with IC_50_ values of 3.5 μM (SI = 10.7) and 3.8 μM (SI = 3.6), respectively [85].

### 6.5. Antibacterial Activity

Compounds **9** (Figure 5), **12**, **15** (Figure 6), **33**, **38**–**40** (Figure 9), **41**, **48** (Figure 10), **57**–**59** (Figure 12), **73** (Figure 14) and **77** (Figure 15) were investigated against 30 human pathogens, including 27 clinical strains and three isolates of marine origin. Interestingly, only **33** and **41** were active against Gram-positive bacteria, belonging to the *Staphylococcus* and *Enterococcus* genera. Compounds **33** and **41** were also found to inhibit the ATP production in purified mammalian rod outer segments, which is associated directly or indirectly with various human diseases [52].

In order to facilitate readers to quickly localize biological and pharmacological activities of these uncommon terpenoid compounds from the genus *Salvia*, the class of compounds (including compound names and numbers), plant sources, part used and biological/pharmacological activities are summarized in Table 1.

## 7. Conclusions and Future Perspectives

The outlined examples highlight that plants of the genus *Salvia* are an interesting source for compounds with novel and unique scaffolds for further development as drug leads. From the initial breakthrough, the structures of uncommon *Salvia* terpenoids, especially the dammarane-type triterpenoids and sesterterpenoids, have not been thoroughly investigated. Although this review covers 106 terpenoids from members of the genus *Salvia* and some proposed biosynthetic pathways, biological and pharmacological activities have been less considered due to the shortcoming in information regarding the biological properties obtained from previous studies. The biosynthetic pathways for apiananes, hassananes, and dammaranes indicate that secondary metabolite production can be species-specific. Owing to huge potential of underexplored bioactivities, terpenoids from *Salvia* species can certainly be explored as a promising group of secondary metabolites for applications in the pharmaceutical, cosmeceutical and agro industries. On the other hand, due to a lack of reliable technology, the relative and absolute configurations of many terpenoids studies in the 1970s and 1980s are still undetermined and can be a challenging task for researchers in this field. Therefore, a revisit of the stereochemistry of these compounds by modern chiroptical methods is another important aspect to be addressed. All in all, this review can provide an insight for researchers who look for bioactive secondary metabolites from *Salvia* plants with unique and rare scaffolds.

## Figures and Tables

**Table 1:** Uncommon terpenoids from *Salvia* species and their biological/pharmacological activities.

Compounds	Plant Species	Used Part	Biological Activity	Ref.
**C_23_ terpenoids**				
14-hydroxy-7-methoxy-11,16-diketo-apian-8-en-(22,6)-olide (**1**)	*S. apiana* Jeps	Aerial Parts	-	[45]
7-methoxy-11,16-diketo-apian-8,14-dien-(22,6)-olide (**2**)				
13,14-dioxo-11-hydroxy-7-methoxy-hassane-8,11,15-trien-(22,6)-olide (**3**)	*S. apiana* Jeps	Aerial Parts	-	[46]
*rel*-(5*S*,6*S*,7*S*,10*R*,12*S*,13*R*)-7-hydroxyapiana-8,14-diene-11,16-dion-(22,6)-olide (**4**)	*S. officinalis* L.	Leaves	Weak antioxidant activity	[47,86]
*rel*-(5*S*,6*S*,7*R*,10*R*,12*S*,13*R*)-7-hydroxyapiana-8,14-diene-11,16-dion-(22,6)-olide (**5**)			-	
*rel*-(5*S*,6*S*,7*S*,10*R*,12*R*,13*S*)-7-hydroxyapiana-8,14-diene-11,16-dion-(22,6)-olide (**6**)				
Przewalskin A (**7**) and its oxidation derivative (**8**)	*S. przewalskii* Maxim	Aerial parts	Anti-HIV-1 activity	[48]
**Sesterterpenoids**				
(13*E*)-labd-13(14),17(18)-dien-8*α*,16,19-triol (**9)**	*S. tingitana* Etl.,	Aerial parts	Antibacterial and ATP modulation activity	[52]
3*α*,8*α*,13,14-*erythro*-tetrahydroxy-labd-15,17-dien-16,19-olide (**10**)	*S. palaestina* Bentham	Aerial parts	-	[53]
6*α*,8*α*,15(*S*)-trihydroxy-labd-13(14),17-dien-16(*S*),19-olide (**11**)	*S. dominica* L.	Aerial parts	Inhibition of Tubulin Tyrosine ligase	[54]
(13*E*)-8*α*,23-dihydroxy-labd-13(14),17(18)-dien-16,19-olide (**12**)	*S. tingitana* Etl.	Aerial parts	Antibacterial and ATP modulation activity	[52]
Salvisyriacolide (**13**)	*S. syriaca* L.	Aerial parts	-	[55]
6*α*,8*α*,15(*S*),23-tetrahydroxy-labd-13(14),17-dien-16(*S*),19-olide (**14**)	*S. dominica* L.	Aerial parts	Inhibition of Tubulin Tyrosine ligase	[54]
6*α*,8*α*,23-trihydroxy-labd-13(14),17-dien-16(*R*),19-olide (**15**)				
6*α*,15(*S*),23-trihydroxy-labd-8(22),13(14),17-trien-16(*S*),19-olide (**16**)				
6*α*,8*α*,23-trihydroxy-labd-13(14),15,17-trien-16,19-olide (**17**)				
6*α*,8*α*,23,14,15-*threo*-pentahydroxy-labd-13(21),17-dien-16,19-olide (**18**)				
6*α*,8*α*,23,14,15-*erythro*-pentahydroxy-labd-13(21),17-dien-16,19-olide (**19**)				
6*α*,8*α*,13,23,14,15-*threo*-hexahydroxy-labd-17-en-16,19-olide (**20**)	*S. dominica* L.	Aerial parts	Inhibition of Tubulin Tyrosine ligase	[56]
6*α*,8*α*,15(*S*)-trihydroxy-23-*oxo*-labd-13(14),17-dien-16(*S*),19-olide (**21**)	*S. dominica* L.	Aerial parts	Inhibition of Tubulin Tyrosine ligase	[54]
6*α*,8*α*-dihydroxy-23-*oxo*-labd-13(14),17-dien-16(*R*),19-olide (**22**)				
6*α*,15(*S*)-dihydroxy-23-*oxo*-labd-8(22),13(14),17-trien-16(*S*),19-olide (**23**)				
6*α*,8*α*-dihydroxy-23-*oxo*-labd-13(14),15,17-trien-16,19-olide (**24**)				
6*α*,8*α*,14,15-*threo*-tetrahydroxy-23-*oxo*-labd-13(21),17-dien-16,19-olide (**25**)				
6*α*,8*α*,13,14,15-*threo*-23-*oxo*-pentahydroxy-labd-17-en-16,19-olide (**26**)	*S. dominica* L.	Aerial parts	Inhibition of Tubulin Tyrosine ligase	
Salvimirzacolide (**27**)	*S. mirzayanii* Rech. and Esfandieri.	Aerial parts	-	[57]
8*α*,13,14-*threo*-trihydroxy-labd-15,17-dien-16,19-olide-23-oic acid (**28**)	*S. palaestina* Bentham	Aerial parts	-	[53]
8*α*,13,14-*erythro*-trihydroxy-labd-15,17-dien-16,19-olide-23-oic acid (**29**)				
6*α*,8*α*,15(*S*)-trihydroxy-23-carboxy-labd-13(14),17-dien-16(*S*),19-olide (**30**)	*S. dominica* L.	Aerial parts	Inhibition of Tubulin Tyrosine ligase	[54]
6*α*,8*α*-dihydroxy-23-carboxy-labd-13(14),17-dien-16,19-olide (**31**)				
6*α*,8*α*-dihydroxy-23-carboxy-labd-13(14),15,17-trien-16,19-olide (**32**)				
Salvileucolide methyl ester (**33**)	*S. syriaca* L.	Aerial parts	-	[55]
	*S. hypoleuca* Benth.	Aerial parts	-	[58]
	*S. sahendica* Boiss & Buhse	Aerial parts	-	[59]
	*S. lachnocalyx* Hedge	Aerial parts	Cytotoxic activity	[62]
14-hydroperoxy-13(21)-dehydro-SME (**34**)	*S. hypoleuca* Benth.	Aerial parts	-	[58]
13-hydroperoxy-14-ene-SME (**35**)				
13-*epi*-hydroperoxy-14-ene-SME (**36**)				
14,17-cycloperoxy-13(21)-dehydro-SME (**36**)				
6*α*,8*α*,15(*S*)-trihydroxy-23-carboxymethyl-labd-13(14),17-dien-16(*S*),19-olide (**38**)	*S. dominica* L.	Aerial parts	Inhibition of Tubulin Tyrosine ligase	[54]
(4*R*,5*R*,8*R*,9*R*,10*S*,16*R*,13*E*)-8-hydroxy-23-carboxymethyl-labd-13(14),17(18)-dien-16,19-olide (**39**)	*S. tingitana* Etl.	Aerial parts	Antibacterial and ATP modulation activity	[52]
(4*R*,5*R*,6*S*,8*R*,9*R*,10*S*,15*S*,16*S*,13*E*)-8,15-dihydroxy-23-carboxymethyl-labd-13(14),17(18)-dien-16,19-olide (**40**)				
Salvileucolide-6,23-lactone (**41**)	*S. hypoleuca* Benth.	Aerial parts	-	[58]
	*S. lachnocalyx* Hedge	Aerial parts	Cytotoxic activity	[62]
	*S. tingitana* Etl.	Aerial parts	Antibacterial and ATP modulation activity	[52]
15,16-dehydrosalvileucolide-6,23-lactone-*trans*-epoxide (**42**)	*S. hypoleuca* Benth.	Aerial parts	-	[58]
15,16-dehydrosalvileucolide-6,23-lactone-*cis*-epoxide (**43**)				
15,16-dehydrosalvileucolide-6,23-lactone-13,14-bis-*epi*-*trans*-epoxide (**44**)				
14-hydroperoxy-13(21)-dehydro-13,14-dihydro-salvileucolide-6,23-lactone (**45**)				
Salvileucolide (**46**)	*S. tingitana* Etl.	Aerial parts	Antibacterial and ATP modulation activity	[52]
Lachnocalyxolide B (**47**)	*S. lachnocalyx* Hedge	Aerial parts	Cytotoxic activity	[62]
8*α*,15(*S*)-dihydroxy-labd-13(14),17-dien-23,6*α*-16(*S*),19-diolide (**48**)	*S. dominica* L.	Aerial parts	Inhibition of Tubulin Tyrosine ligase	[54]
8*α*-hydroxy-labd-13(14),15,17-trien-6*α*,23-16,19-diolide (**49**)				
8*α*-hydroxy-13-hydroperoxylabd-14,17-dien-19,16;23,26*α*-diolide (**50**)	*S. sahendica* Boiss & Buhse	Aerial parts	-	[63]
23,6*α*-epoxy-labd-8,13(14),17-trien-16(*R*),19-olide (**51**)	*S. dominica* L.	Aerial parts	Inhibition of Tubulin Tyrosine ligase	[54]
15(*S*)-dihydroxy-23,6*α*-epoxy-labd-13(14),17-dien-16(*S*),19-olide (**52**)				
8*α*,15(*S*),23*α*-trihydroxy-23,6*α*-epoxy-labd-13(14),17-dien-16(*S*),19-olide (**53**)				
8*α*,15(*S*)-dihydroxy-23*α*-*O*-ethyl-23,6*α*-epoxy-labd-13(14),17-dien-16(*S*),19-olide (**54**)				
8*α*-hydroxy-23*α*-O-ethyl-23,6*α*-epoxy-labd-13(14),17-dien-16(*R*),19-olide (**55**)				
8*α*,23-dihydroxy-23,6*α*-epoxy-labd-13(14),15,17-trien-16,19-diolide (**56**)				
(4*R*,5*R*,6*S*,8*R*,9*R*,10*S*,16*R*,23*S*,13*E*)-8,23-dihydroxy-23,6-epoxy-labd-13(14),17(18)-dien-16,19-olide (**57**)	*S. tingitana* Etl.	Aerial parts	Antibacterial and ATP modulation activity	[52]
(13*E*)-8*α*-hydroxy-23*α*-*O*-methyl-23,6*α*-epoxy-labd-13(14),17(18)-dien-16,19-olide (**58**)				
(4*R*,5*R*,6*S*,8*R*,9*R*,10*S*,15*S*,16*S*,23*S*,13*E*)-8,15-dihydroxy-23-*O*-methyl-23,6-epoxy-labd-13(14),17(18)-dien-16,19-olide (**59**)				
Salvidominicolide B (**60**)	*S. dominica* L.	Whole parts	-	[64]
13-*epi*-salviaethiopisolide (**61** and **61′**)	*S. aethiopis*	Aerial parts	-	[65]
Salviaethiopisolide (**62** and **62′**)				
Yosgadensolide A (6*α*,14-dihydroxymanoyloxide-15,17-dien-16,19-olide; **63**)	*S. yosgadensis* Freyn et Bornm.	Aerial parts	-	[66]
yosgadensolide B (6*α*,16-dihydroxymanoyloxide-14,17-dien-16,19-olide; **64**)				
3*β*-hydroxymanoyloxide-14(*E*),17-dien-16-*oxo*-19-oic acid (**65**)	*S. aethiopis* L.	Aerial parts	-	[67]
Hydroxymanoyloxide-14,17-dien-16-*oxo*-19-oic acid (**66**)				
Hydroxymanoyloxide-14,17-dien-16-*oxo*-19,23-dioic acid (**67**)				
(4*R*,5*R*,8*R*,9*R*,10*S*,13*S*,14*S*)-14-hydroxymanoyloxide-15,17-dien-15(*Z*)-16,19-olide (**68**)	*S. mirzayanii* Rech. and Esfandieri.	Aerial parts	-	[68]
(4*R*,5*R*,8*R*,9*R*,10*S*,13*R*,14*S*)-14-hydroxymanoyloxide-15,17-dien-15(*Z*)-16,19-olide (**69**)				
(4*R*,5*R*,8*R*,9*R*,10*S*,13*S*,14*R*)-14-hydroxymanoyloxide-15,17-dien-15(*Z*)-16,19-olide (**70**)				
(4*R*,5*R*,8*R*,9*R*,10*S*,13*R*,16*R*)-14-hydroxymanoyloxide-17-en-16,19-olide (**71**)				
(4*R*,5*R*,8*R*,9*R*,10*S*,13*R*)-manoyloxide-15,17-dien-15(*Z*)-16,19-olide (**72**)				
(14*E*)-methylmanoyloside-14,16,18-trien-16,19-oxide-23-carboxilate (**73**)	*S. tingitana* Etl.	Aerial parts	Antibacterial and ATP modulation activity	[52]
Lachnocalyxolide A (**74**)	*S. lachnocalyx* Hedge	Aerial parts	Cytotoxic activity	[62]
Lachnocalyxolide C (**75**)				
Salvidominicolide A (**76**)	*S. dominica* L.	Whole parts	-	[64]
(13*E*)-4*α*,6*α*,8*α*-trihydroxy-labd-13(14),17(18)-dien-16,19-olide (**77**)	*S. tingitana* Etl.	Aerial parts	Antibacterial and ATP modulation activity	[52]
Yosgadensonol (**78**)	*S. yosgadensis* Freyn et Bornm.	Aerial parts	-	[69]
13-*epi*-yosgadensonol (**79**)				
6-dehydroxy-yosgadensonol (**80**)	*S. limbata* C. A. Meyer	Aerial parts	-	[70]
6-dehydroxy-13-*epi*-yosgadensonol (**81**)				
(17,18,19,20-tetranor-13-*epi*-manoyloxide-14-en-16-oic acid-23,6*α*-olide; **82**)	*S. sahendica* Boiss & Buhse	Aerial parts	-	[63]
**Dammarane triterpenoids**				
Salvilymitol (**83**)	*S. hierosolymitana* Boiss.	Aerial parts	-	[73]
Salvilymitone (**84**)				
Pixynol (**85**)	*S. barrelieri* Ettling	Roots	Weak antioxidant activity	[74,75]
(20*S*,24*R*)-epoxydammar-12*β*,25-diol-3-one (**86**)	*S. bicolor*	Whole parts	-	[76]
Santolin A (**87**)	*S. santolinifolia* Boiss.	Whole parts	-	[77]
Santolin B (**88**)				
Santolin C (**89**)				
Amblyol (**90**)	*S. aspera* M. et G.	Aerial parts	-	[79]
Amblyone (**91**)				
Russelliinoside A (**92**)	*S. Russellii* Beneth.	Aerial parts	Cytotoxic activity	[80]
Russelliinoside B (**93**)				
Russelliinoside C (**94**)				
**Uncommon triterpenoids**				
Salvadione A (**95**)	*S. bucharica* M. Pop	Aerial parts	-	[81]
Salvadione B (**96**)				
Salvadiol (**97**)	*S. bucharica* M. Pop	Aerial parts	-	[82]
Salvadione C (**98**)	*S. hydrangea* DC. ex. Benth.	Aerial parts	Antiparasitic activity	[83]
Perovskone B (**99**)				
Hydrangenone (**100**)	*S. hydrangea* DC. ex. Benth.	Aerial parts	Antiparasitic activity	[84]
Salvadione A (**95**)	*S. hydrangea* DC. ex. Benth.	Aerial parts	Antiparasitic activity	[85]
Hydrangenone B (**101**)				
Pervoskones C (**102**)				
Pervoskones D (**103**)				
Pervoskones E (**104**)				
Pervoskones F (**105**)				
Salvadione D (**106**)				

## Data Availability

Data sharing is not applicable to this article.
